# Mammal-exclusion fencing improves the nesting success of an endangered native Hawaiian waterbird

**DOI:** 10.7717/peerj.10722

**Published:** 2021-03-01

**Authors:** Dain L. Christensen, Kristen C. Harmon, Nathaniel H. Wehr, Melissa R. Price

**Affiliations:** Department of Natural Resources and Environmental Management, College of Tropical Agriculture and Human Resources, University of Hawaii at Mānoa, Honolulu, Hawaii, United States of America

**Keywords:** Conservation, Invasive Predators, Exclusion Fencing, Nesting Success, Hawaiian Stilt, Waterbirds

## Abstract

Invasive predator control is often critical to improving the nesting success of endangered birds, but methods of control vary in cost and effectiveness. Poison-baiting or trapping and removal are relatively low-cost, but may have secondary impacts on non-target species, and may not completely exclude mammals from nesting areas. Mammal-exclusion fencing has a substantial up-front cost, but due to cost savings over the lifetime of the structure and the complete exclusion of mammalian predators, this option is increasingly being utilized to protect threatened species such as ground-nesting seabirds. However, non-mammalian predators are not excluded by these fences and may continue to impact nesting success, particularly in cases where the fence is designed for the protection of waterbirds, open to an estuary or wetland on one side. Thus, there remains a research gap regarding the potential gains in waterbird nesting success from the implementation of mammal-exclusion fencing in estuarine systems. In this study, we compared the nesting success of endangered Hawaiian Stilts (Ae‘o; *Himantopus mexicanus knudseni*) within a mammal-exclusion fence to that of breeding pairs in a nearby wetland where trapping was the sole means for removing invasive mammals. We predicted success would be greater for breeding pairs inside the exclusion fence and the hatchlings inside the enclosure would spend more time in the nesting area than hatchlings at the unfenced site. During a single breeding season following construction of a mammal-exclusion fence, we used motion-activated game cameras to monitor nests at two sites, one site with mammal-exclusion fencing and one site without. Clutch sizes and hatch rates were significantly greater at the fenced site than the unfenced site, but time spent by chicks in the nesting area did not differ between sites. These results add to the mounting body of evidence that demonstrates the effectiveness of mammal-exclusion fencing in protecting endangered birds and suggests it can aid endangered Hawaiian waterbirds toward recovery. These results also suggest that the single greatest predatory threat to the Hawaiian Stilt may be invasive mammals, despite a host of known non-mammalian predators including birds, crabs, turtles, and bullfrogs, as the complete exclusion of mammals resulted in significant gains in nesting success. As additional fences are built, future studies are necessary to compare nesting success among multiple sites and across multiple seasons to determine potential gains in fledging success and recruitment.

## Introduction

Endangered species often face multiple threats, leaving decision-makers with the task of discerning which set of actions is most likely to achieve recovery, given budget constraints (Beyer et al., 2018; Carwardine et al., 2019). Further, potential actions to abate a given threat often vary in effectiveness and cost resulting in further decision complexity (Tulloch et al , 2015). Managers may improve outcomes for future decisions by monitoring the response of protected species following conservation interventions (Runge, 2011). However, few studies utilize monitoring data to implement adaptive management in a structured manner (Williams, Balmford & Wilcove, 2020). A critical evaluation of predictions made prior to taking action, in light of actual outcomes, can improve our understanding regarding how a system works (Moon et al., 2019). This process is particularly important when there are multiple options available to abate a given threat, each with varying costs and impacts.

Invasive predators, particularly mammals, contribute to native bird declines and species extinctions worldwide (Doherty et al., 2016). Ground-nesting birds, in particular, tend to benefit the most from control of these predators (Holt et al., 2008; Lavers, Wilcox & Donlan, 2010). The task of managing invasive mammalian predators is currently approached by poison-baiting, trapping and removal, and/or exclusion fencing (O’Donnell, Clapperton & Monks, 2014). Poison-baiting is the most cost-effective method of predator removal over a large area, but this option may impact non-target species and often carries a high social cost (Howald et al., 2005; Duron, Shiels & Vidal, 2017). Trapping and removal, as well as exclusion fencing, minimize secondary impacts to non-target species. However, trapping and removal efforts may be ineffective if there is increased predator fecundity or survival, rapid immigration from adjacent sites, or if too few predators are removed to have a detectable benefit (Côté & Sutherland, 1997; Meckstroth & Miles, 2005). Furthermore, removal of a single predator type, such as feral cats (*Felis catus*), may lead to the release of meso-predators, such as rats (*Rattus spp.*), thereby increasing predation rates (Rayner, Hauber & Clout , 2007). Thus, exclusion of a diversity of predator types by exclusion fencing may be more effective in protecting vulnerable species (Smith et al., 2010).

Exclusion fences serve an important role in supporting the recovery of some species (Smith et al. , 2011; Innes et al., 2012) including passerines, seabirds, and shorebirds (Neuman et al., 2004; Sanders, Brown & Keedwell, 2007; Rickenbach et al., 2011; Malpas et al. , 2013; Young et al., 2013). Successful implementation is heavily influenced by design and placement. Managers may strategically use natural barriers such as cliffs, peninsulas, and bodies of water to reduce costs and improve results. However, these designs may be more susceptible to mammalian incursions as these designs are not fully encircled (Young et al. , 2012). Additionally, enclosures that use bodies of water as a natural barrier may still be accessible to amphibious and aquatic predators, and most enclosure designs are vulnerable to avian predators. While many studies have evaluated different predator control methods, no study to date has evaluated the effectiveness of exclusion fencing in protecting endangered waterbirds nesting in coastal environments.

The Hawaiian Stilt (Ae‘o, *Himantopus mexicanus knudseni*), a sub-species of the Black-necked Stilt (*Himantopus mexicanus*), declined in the 19th century due to habitat loss and degradation, over-harvesting, and the introduction of invasive predators (USFWS, 2011). Following listing as endangered by the U.S. Fish and Wildlife Service (USFWS) in 1970, state and federal efforts have established over 6,000 hectares of designated habitat (Reed et al., 2011; USFWS, 2011). Predator removal efforts vary by site and re-incursions by feral cats, rats, and mongoose (*Herpestes javanicus*) from nearby areas are common. Indeed, introduced mammalian predators remain a major threat to Hawaiian Stilt nesting success, even within state and federally managed wetlands (Harmon Wehr & Price, 2020). In 2011 the population was estimated at ∼1,500 individuals, with a slowly increasing trend (Reed et al., 2011; Reed et al, 2015). However, habitat availability may be a limiting factor in achieving recovery (Reed, Elphick & Oring , 1998) and is likely to decrease with sea level rise (Harmon et al. 2020b). Thus, it is particularly important that reproductive success in available habitat is maximized.

Prior to the introduction of invasive predators, native predators of the Hawaiian Stilt were limited to avian species such as the Hawaiian Short-eared Owl (Pueo; *Asio flammeus sandwichensis*), the Hawaiian Hawk (I‘o; *Buteo solitarius*), the now extinct Stilt Owl (*Grallistrix spp.*), as well as other native waterbirds such as the Black-crowned Night Heron (Auku‘u; *Nycticorax nycticorax*). Today, a plethora of non-native predators from a variety of taxonomic groups prey on adult stilts, chicks, and eggs (Underwood, 2013; Harmon Wehr & Price, 2020). Avian predators predominantly use visual cues to locate potential prey, whereas nocturnal predators, such as mammals, may rely on olfaction (Conover, 2007). Hawaiian Stilts have developed a few anti-predator behaviors such as broken wing displays and aggressive “dive-bombing.” Although these behaviors are largely efficient in deterring avian predators (Sordahl, 2004), Hawaiian Stilts also remove eggshells from the nest after chicks have hatched to reduce scent, an antipredator behavior typical of North American Recurvirostrids (Sordahl, 1994). Soon after the brood hatches, adults lead their young away from the nesting area (Colemann, 1981), possibly in search of better foraging or to evade a lingering scent that may attract potential predators, or both. Further, Hawaiian Stilts have the capacity to re-nest, combine egg clutches, and aggressively respond to potential predators, suggesting they may be resilient against a variety of predators, similar to Black-necked Stilts (Colemann, 1981; Sordahl, 2004).

In this study we compared the nesting success of Hawaiian Stilts in a wetland protected by a mammal-exclusion fence and a regular trapping regime to a nearby wetland without an exclusion fence, where mammals were removed solely via trapping. We predicted nesting success would be greater for breeding pairs inside the exclusion fence and the hatchlings would spend more time in the nesting area compared to the site where mammals were controlled via trapping and removal only.

## Methods

### Study area

**Figure 1:**
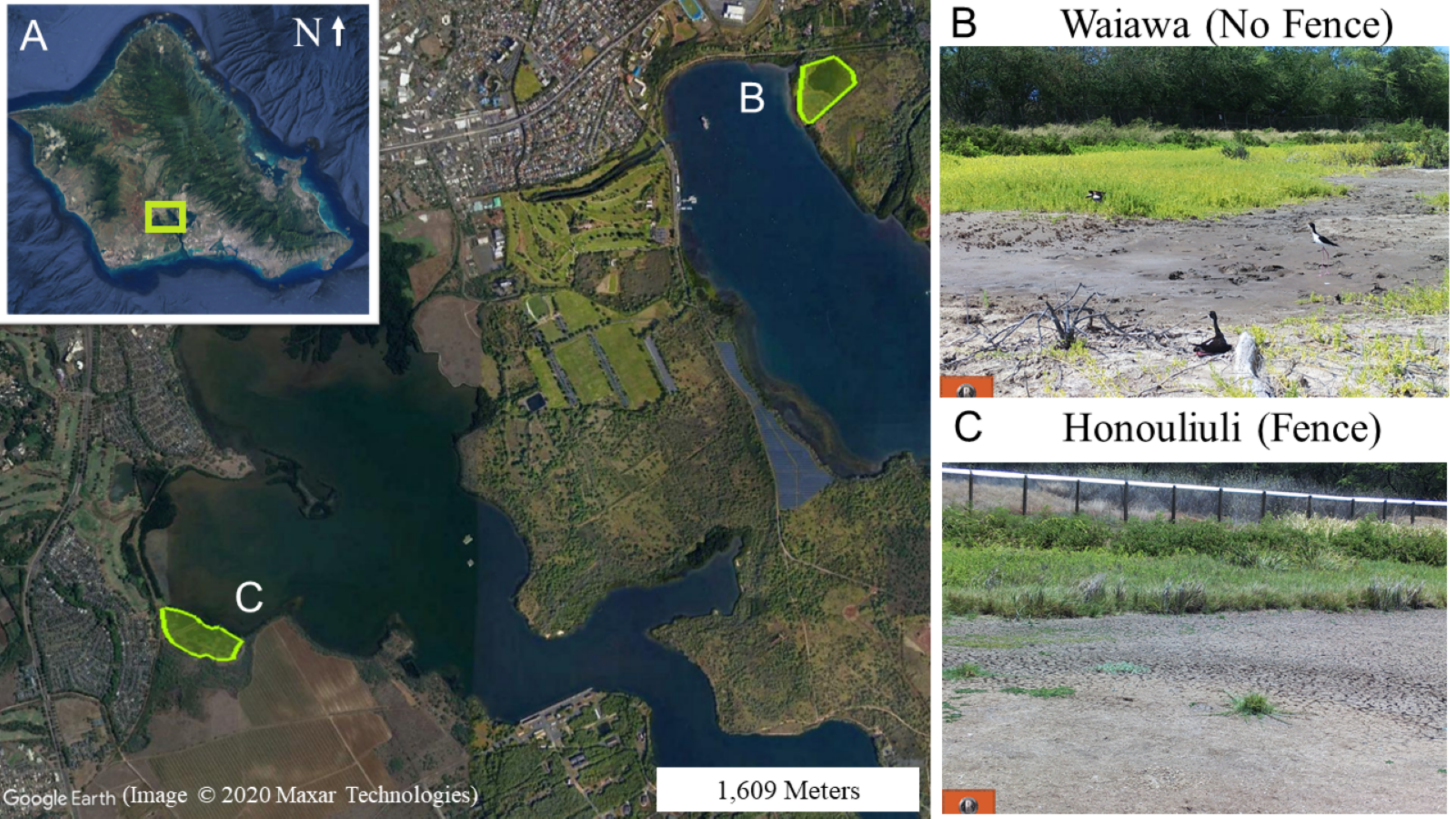
(A) The island of O‘ahu with Pearl Harbor National Wildlife Refuge (PHNWR) study sites at (B) Waiawa and (C) Honouliuli. Google Earth image, ©2020 Maxar Technologies.

**Figure 2:**
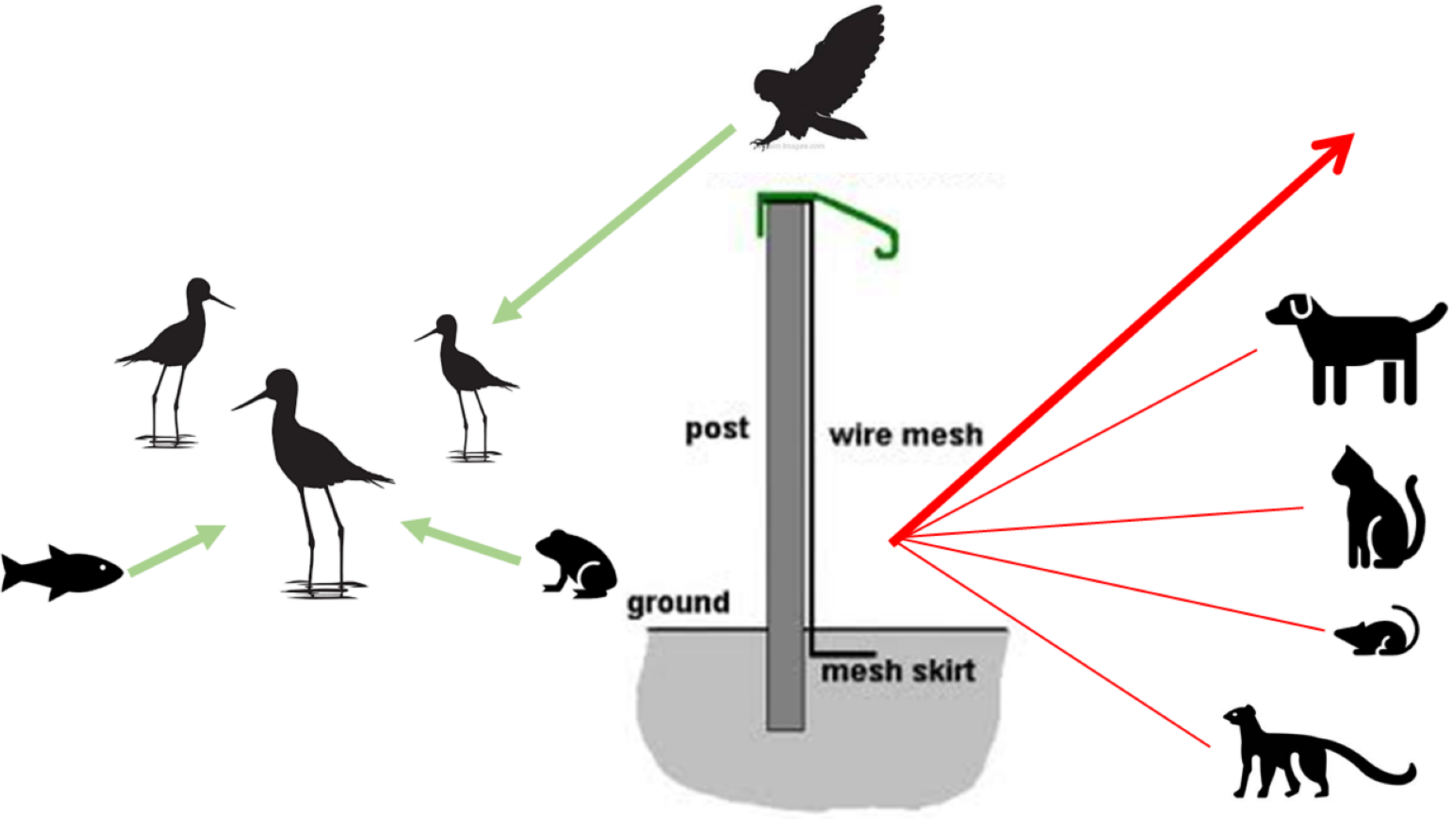
Conceptual model of mammal-exclusion fencing and threat dynamics of potential predators from different taxonomic groups. The fence at the Honouliuli wetland unit stands 2 m tall and has a recurved hood that extends out 330 mm to prevent mammals from climbing over. The 6 mm ×25 mm stainless steel mesh prevents smaller species from squeezing through and is resistant to corrosion. A skirt extends underground to prevent digging.

**Figure 3:**
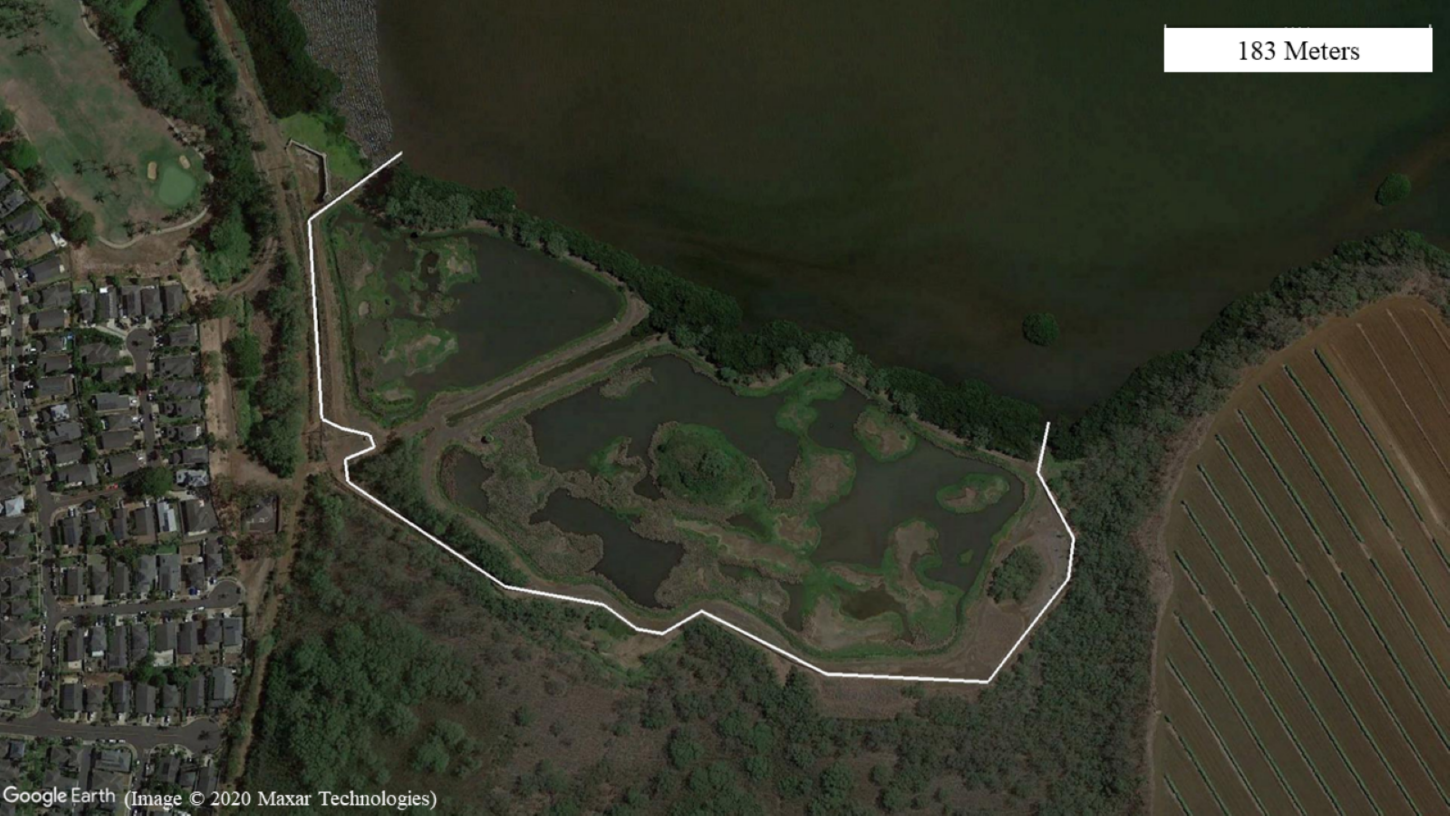
Honouliuli wetland unit with the white line representing the course of the fence and the two ends extending into the water at Pearl Harbor. Google Earth image, ©2020 Maxar Technologies.

**Figure 4:**
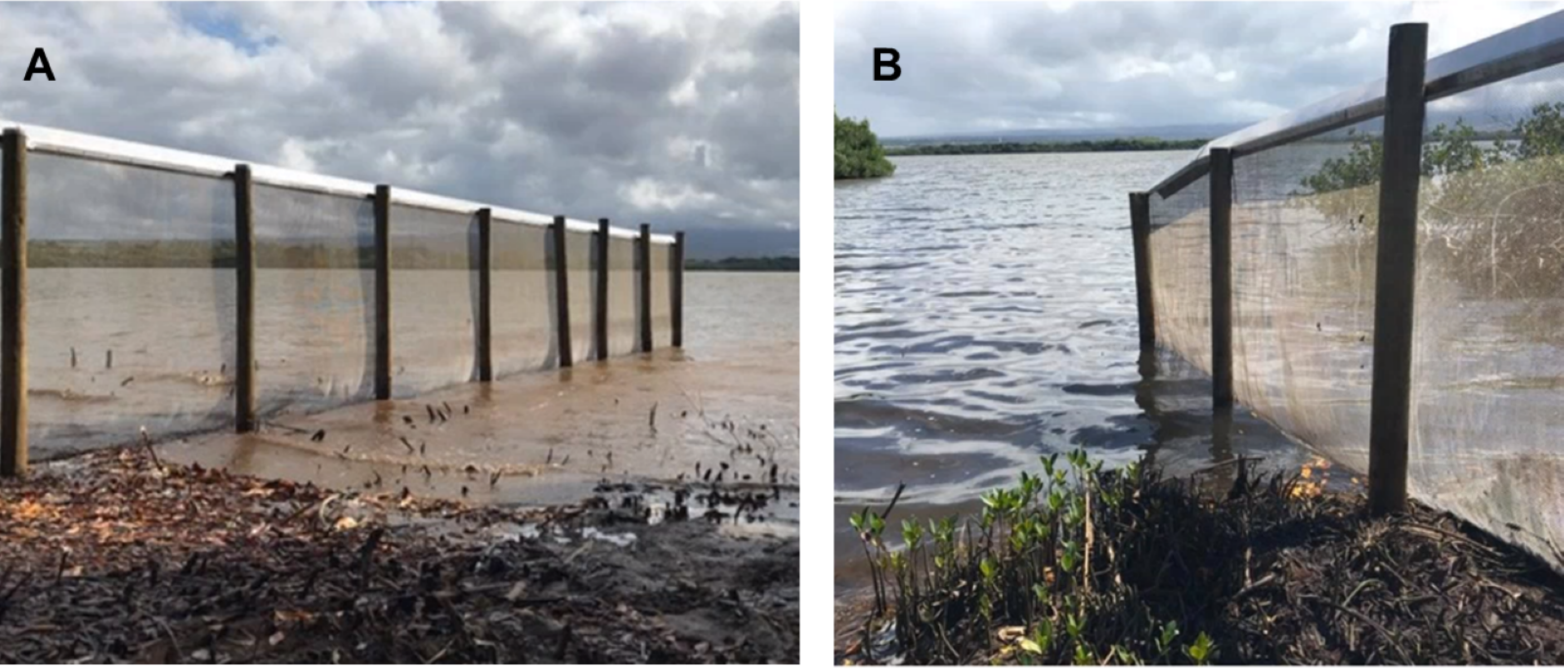
The west (A) and east (B) ends of the exclusion fence extending into Pearl Harbor, using the water as a natural barrier to exclude mammalian predators. Photos were taken within an hour of each other on the same day by Lindsay Young at medium tide.

This study was conducted at two wetland units, Honouliuli and Waiawa (Fig. 1), both part of the larger Pearl Harbor National Wildlife Refuge (PHNWR) complex. PHNWR was established in 1976 as mitigation for the construction of the Honolulu International Airport Reef Runway (USFWS, 2009). In August 2018, the USFWS completed a 1,006 m mammal-exclusion fence (Fig. 2) around the Honouliuli wetland unit (Fig. 3), in which one side is open to an estuary (Fig. 4) and is based on the Xcluder™ Kiwi model designed for multiple-species exclusion (Day & MacGibbon, 2007). The two sites are in the same region on the island of O‘ahu with similar proximity to urban development and similar rainfall regimes ranging from 555–619 mm annually (Giambelluca, Shuai & Businger, 2014). The Honouliuli unit (hereafter referred to as ‘fenced site’; 14.7 hectares) is typically less saline than the Waiawa unit (hereafter referred to as ‘unfenced site’; 9.9 hectares; Colemann, 1981). Plant community composition differs somewhat between sites with water hyssop (‘Ae‘ae ; *Bacopa monnieri*), non-native pickleweed (*Batis maritima*), and cattails (*Typha spp.*) dominating the fenced site, while the unfenced site is dominated almost exclusively by non-native pickleweed and Guinea grass (*Megathyrus maximus*) along the perimeter.

**Table 1:** Potential predators of the Hawaiian Stilt and the life stage they depredate.

**Common Name**	**Scientific Name**	**Eggs**	**Chicks**	**Reference**
Barn Owl^a^	*Tydo alba*		x	Robinson et al. (1999)
Black-crowned Night Heron	*Nycticorax nycticorax*	x	x	Colemann (1981)
Bullfrog^a^	*Rana catesbeiana*		x	USFWS (2009)
Cattle Egret^a^	*Bubulcus ibis*	x	x	Colemann (1981)
Common Myna^a^	*Acridotheres tristis*	x	x	Colemann (1981)
Domestic Cat^a^^b^	*Felis catus*	x	x	Colemann (1981)
Domestic Dog^a^	*Canis familiaris*	x	x	Colemann (1981)
Hawaiian Short-eared Owl	*Asio flammeus sandwichensis*	x	x	Colemann (1981)
Indian Mongoose^a^	*Herpestes javanicus*	x	x	Colemann (1981)
Large fish	–		x	USFWS (2011)
Laughing Gull	*Larus atricilla*	x		Colemann (1981)
Pig^a^	*Sus scrofa*	x		Underwood et al., (2014)
Rat^a^^b^	*Rattus spp.*	x		Colemann (1981)
Ruddy Turnstone	*Arenaria interpres*	x		Colemann (1981)

**Notes.**

a Indicates a non-native introduced species.

b Indicates potential predators confirmed in this study.

Both sites are managed by the USFWS, with similar predator communities prior to the construction of the fence (Table 1). Trapping by USFWS personnel occurred regularly at both sites between 2018 and 2020. USFWS used Tomahawk style live traps meant to target rats, feral cats, and mongoose as well as DOC 250 traps that primarily target rats and mongoose. The live traps were opened on Mondays, checked on Wednesdays and closed on Fridays. Traps were baited with Koi food, cat food, salmon oil, sardines, Vienna sausage, and/or clam juice and targeted rats, feral cats, mongoose, and mice. The unfenced site had a combination of 16 DOC 250 traps and 13 live traps while the fenced site had nine DOC 250 traps and seven live traps (Figs. S1 & S2).

### Data collection

This study was conducted under permits obtained from the U.S Fish and Wildlife Service (SUP#2019-006 and #TE-25955C-2), the State of Hawaii Department of Land and Natural Resources, Division of Forestry and Wildlife (WL-1910), and was approved by the University of Hawaii at Mānoa Institutional Animal Care and Use Committee (#17-2733-2). From March until August 2019, active nests were identified by surveying for incubating adults. A survey consisted of driving around the managed area and spotting incubating adults with binoculars or field scopes. Once incubating adults were identified, approximate locations were noted, and nests were located on foot. For each nest, GPS coordinates were recorded and a Bushnell HD No-Glow Trophy Camera (Bushnell Corporation, Overland Park, KS, USA) was placed 3 m from the nest in accordance with USFWS permit requirements, mounted on a pole 1.2 m off the ground, and pointed downward for a direct view of the nest and some of the surrounding area. The *nesting area* was defined by the camera’s field of view meant to capture the nest and the immediate area around the nest (Figs. S3 & S4). Cameras were set as soon as the nest was discovered and programmed to take one photo every five minutes using field scan mode, one photo after being motion-triggered, and another photo following a two-second delay after the initial trigger. Cameras were checked once or twice per week to maintain memory and battery power. All nests that had at least a single egg hatch were monitored for a minimum of 10 days after hatch or until the adult(s) discontinued incubation and abandoned un-hatched eggs, whichever came last. All photos were processed using VirtualDub open source software (VirtualDub, 2013). Based on camera data, we recorded the number of eggs laid, the number of eggs hatched, the time spent by chicks in the nesting area, and predation events.

The USFWS collected data on the species trapped for each site and the residual relative abundance via tracking tunnels. Tracking indices were collected once in 2018, twice in 2019 and once again in 2020. An index of relative abundance was calculated for the unfenced site as well as inside and outside the fenced site. Species specific trapping data were compiled by USFWS and summarized into annual captures for each site. All trapping and tracking tunnel data prior to 2018 were collected by other agencies and those records were not retained.

### Statistical analysis

All analyses were performed in the program R 3.5.1. (R Core Team, 2019). Packages included *plyr* (*v1.8.4*; Wickham, 2011), *dplyr* (*v0.8.3* Wickham etal, 2019), *Rmisc* (*v1.5*; Hope, 2013), *ggplot2* (*v3.2.1*; Wickham, 2016), and *car* (*v3.0.3*; Fox & Weisberg, 2019)*.* Welch’s *t*-tests correct for unequal variances between groups and were used to compare outcomes between sites, including the number of eggs laid per nest, the number of eggs hatched per nest, the proportion of eggs hatched per nest between sites, and the time spent by chicks in the nesting area. Because the chicks could not be identified at the individual level, the brood was measured together as a collective unit and their time spent in the nesting area was measured from the first hatch to the last time one of the hatchlings was in the frame. As Hawaiian Stilts often renest after unsuccessful nesting attempts (Colemann, 1981), any differences in clutch size between the two sites may be attributed to renesting. Thus, we used a Welch’s *t*-test to test for differences in clutch size between early and late nests at the unfenced site. Our dataset has a convenient ‘natural’ break between May 8–20th for distinguishing early and late nesters. Early nesters ranged from 25-Mar-2019–08-May-2019 while late nesters ranged from 20-May-2019–04-Jul-2019. Within each site, paired *t*-tests were used to compare the number of eggs laid with the number of eggs hatched for each nest. Values are reported as mean plus or minus standard error ($\bar {X}$ ±  SE). Tracking tunnel indices were calculated by USFWS following established protocol (USFWS, 2009). No further analyses were performed on USFWS trapping and tunnel tracking data due to data gaps and low sample size. These data should be viewed as descriptive.

## Results

At the unfenced site, 37 nests were discovered (3.73 nests/ha), compared with 18 total nests discovered inside the mammal-exclusion fence (1.22 nests/ha). Of the discovered nests, 21 of 37 (57%) at the unfenced site, and 9 of 18 (50%) at the fenced site, had useable camera data that spanned egg laying through hatching, and therefore were included as ‘observed nests’ in this study. The remaining nests were either discovered too late in the nesting cycle, or suffered a battery failure/memory malfunction resulting in a lack of data and exclusion from analyses.

**Figure 5:**
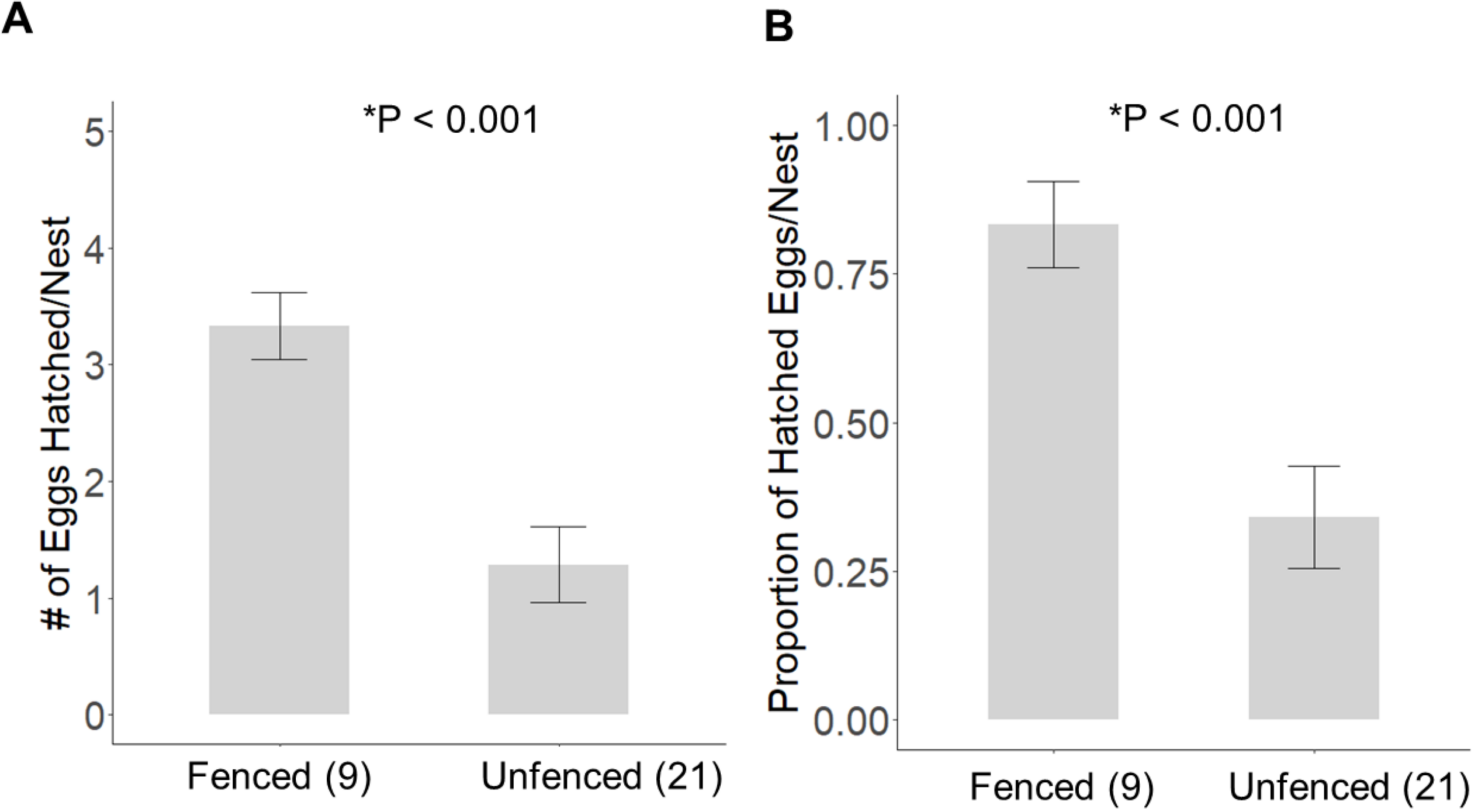
The average (±SE) number of eggs hatched (A) and the proportion of eggs hatched (B) per observed nest at each site for 2019.

**Figure 6:**
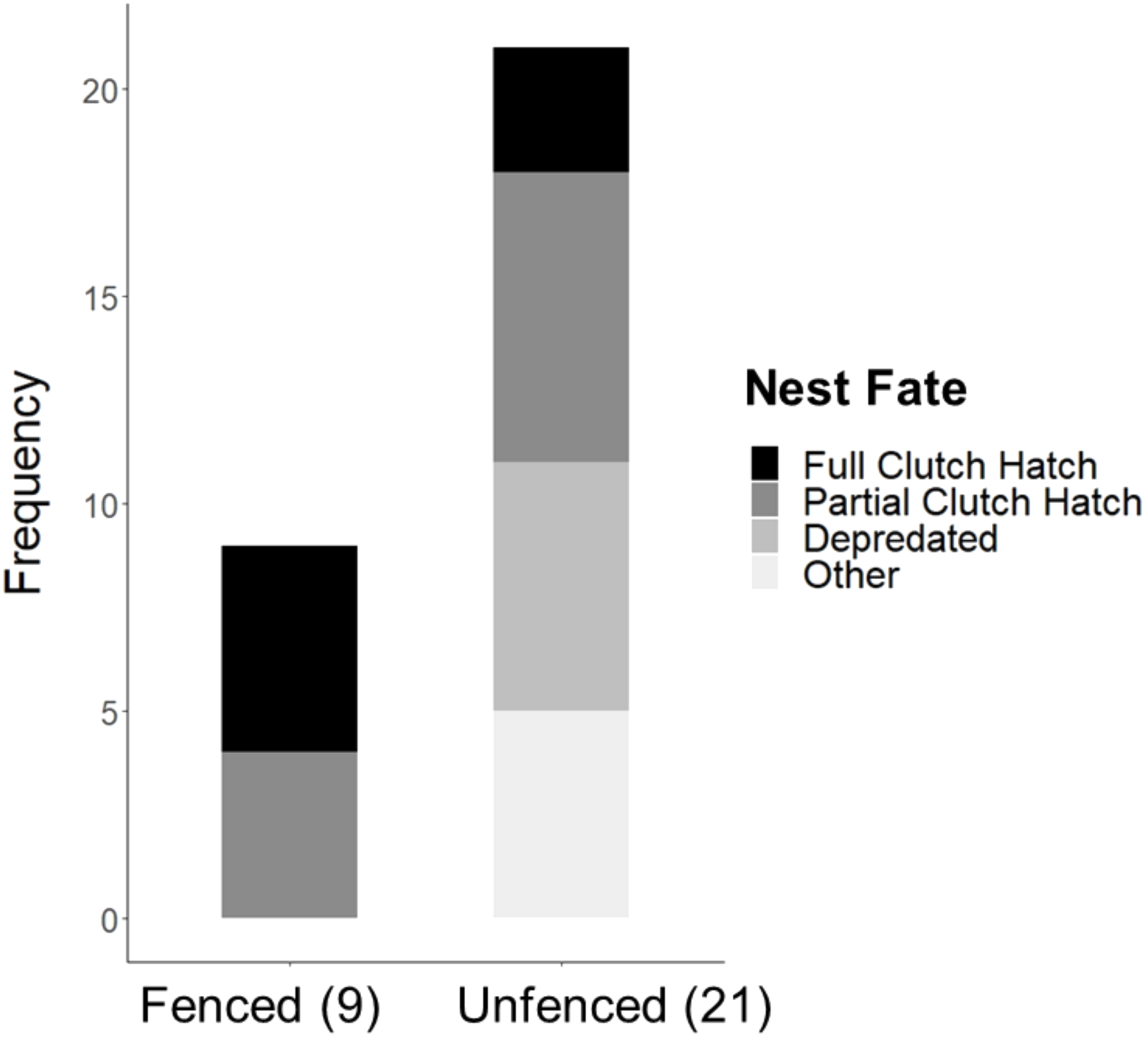
Observed Hawaiian Stilt nest fates at the fenced (9) and unfenced (21) sites. The “Other” category consists of flooded, abandoned, and unknown fates.

Significantly more eggs were laid per nest at the fenced site (4.00 ±  0.00; *t*_20_ = 3.16, *P* = 0.005), compared with the unfenced site (3.67 ±  0.11). At both sites, the number of eggs hatched per nest was significantly less than the number of eggs laid (fenced: *t*_8_ = 2.31, *P* = 0.050; unfenced: *t*_20_ = 7.63, *P* < 0.001); however, a significantly greater number of eggs hatched per nest at the fenced site (3.33 ±  0.29; *t*_25_ = 4.71, *P* <  0.001; Fig. 5) compared to the unfenced site (1.29 ±  0.33). At the fenced site, none of the observed nests were depredated and all nests had at least one egg hatch (*n* = 9). Further, five of the nine nests (55.6%) hatched the full clutch. At the unfenced site, 10 of 21 nests (47.6%) had at least one egg hatch and six of 21 (28.6%) were depredated, three by a feral cat, two by rats and the sixth fell prey to an unknown predator. Five nests (23.8%) were abandoned, flooded, or had unknown fates (Fig. 6). Three of 21 (14.3%) nests at the unfenced site hatched the full clutch . At the unfenced site, clutch sizes did not differ between early (3.70 ±  0.15, *n* = 10) and late nesters (3.6 ±  0.16, *n* = 10; *t*_18_ = 0.45*, P* = 0.66*)*.

Seven nests at the fenced site and nine nests at the unfenced site had usable data to test time spent by chicks in the nesting area following hatching. There was no difference between sites in the time spent by the stilt chicks in the nesting area after hatching (*t*_14_ = 0.59, *P* = 0.56; Fig. S5). During the 2019 study period, potential predators detected by our cameras at the fenced site included the Black-crowned Night Heron, Cattle Egrets (*Bubulcus ibis*), Common Mynas (*Acridotheres tristis*), and Bullfrogs (*Rana catesbeiana*). At the unfenced site, in addition to the species observed at the fenced site, potential predators detected included rats, feral cats, and the small Indian mongoose. Defensive behaviors were observed at each site toward bullfrogs, Black-crowned Night Herons, and Common Mynas. Defensive behaviors included barking, mobbing, dive-bombing, and aerial chasing.

Inside the fenced site between 2018 and 2020 only one feral cat was trapped. Meanwhile, 26 feral cats were trapped within the unfenced site during the same time period (Table 2). Both rats and mongoose were detected and/or trapped at both sites from 2018 to 2020 (Figs. S6–S8).

**Table 2:** Total number of individuals caught per year for each target species at each site. ”Other” represents all other captures that were not feral cats, rats or mongoose. The capture period for both sites spanned 30-Apr-2018–19-Dec-2018, 28-Jan-2019–30-Dec-2019, and 02- Jan-2020– 09-Oct-2020. A government shutdown occurred between 22-Dec- 2018–25-Jan-2019 resulting in over a month where trapping did not occur.

**Year**	**Site**	**Feral Cat**	**Mongoose**	**Rat**	**Other**
2018	Fenced	1	28	11	23
2019	Fenced	0	88	14	84
2020	Fenced	0	21	11	22
2018	Unfenced	8	111	12	11
2019	Unfenced	7	149	5	44
2020	Unfenced	11	114	7	103

## Discussion

As Hawaiian waterbird nesting habitat is likely to decline due to sea level rise (Harmon et al., 2020b), conservation actions maximizing reproductive success in remaining habitat are necessary to achieve recovery. Prior to this study, the effectiveness of mammal-exclusion fencing versus trapping of mammals alone had not been evaluated. Within the first nesting season after construction of a mammal-exclusion fence at a federally protected wetland, we found that nesting success was significantly higher for Hawaiian Stilts nesting inside the fence, compared with those nesting in a nearby wetland where trapping and removal of mammalian predators was the sole means of predator control. Unexpectedly, a significantly higher number of eggs were laid per nest at the fenced site compared with the unfenced site. Together, this resulted in nearly three times the number of eggs hatched per nest inside the mammal-exclusion fence, compared with nests at the site with trapping alone. Thus, mammal-exclusion fencing may play an important role in maximizing reproductive success for Hawaiian waterbirds, as sea level rise decreases available nesting habitat.

Importantly, our results suggest that mammalian predators are the greatest threat to hatching success in Hawaiian Stilts, as the complete exclusion of mammals from the nesting area resulted in substantial gains in the number of eggs laid and chicks hatched per nesting pair. Potential predators such as herons, owls, crabs, turtles, and bullfrogs were not excluded by the fence, and thus were expected to potentially impact nesting success at both sites. Despite detection of these predator types inside the mammal-exclusion fence, they were not observed depredating eggs, potentially due to defensive behaviors such as mobbing, dive-bombing, or others typical of Black-necked Stilts (Sordahl, 2004). Anecdotally, on several occasions, stilt pairs were observed engaging with Black-crowned Night Herons well out of the frame of the camera. Stilts may engage with different predators at different distances (Sordahl, 2004) and any interactions out of the frame of the camera could not be recorded consistently. Further, as detections by motion-activated cameras are imperfect, cameras may have missed predation events or visits to the nests by potential predators.

This study did not track fledging success of the stilts and potential predators may have impacted mortality of chicks at a later stage, hindering recruitment. Further research is needed that evaluates the impact of exclusion fencing on fledging success in Hawaiian Stilts, as well as parental and chick behaviors inside and outside of mammal-exclusion fencing. Differences in water salinity, food availability, and other factors not considered here may also have influenced nesting success. If other factors do not impact fledging success and juvenile survival prior to recruitment, we may expect increased nesting success to result in increased recruitment and larger breeding populations (Smith et al. , 2011).

Our 2019 camera trap data corroborates the USFWS tracking tunnel and trapping data for the same year. Although USFWS staff visit both sites when checking traps, the unfenced site had more traps deployed between 2018 and 2020 resulting in greater trapping effort or more ‘trap nights’ at the unfenced site, potentially explaining why more feral cats and mongoose were trapped at the unfenced site. Regardless, these data do suggest smaller populations of feral cats and mongoose exist at the fenced site. Future studies are needed to compare the cost-effectiveness of different trapping levels with that of exclusion fencing.

Mammal-exclusion fencing may decrease physiological stress in nesting birds induced by the need to remain vigilant for predators, thereby increasing reproductive success. A decreased need for vigilance has been associated with larger clutch sizes and increased fitness in other species (Zanette et al., 2011; Thomson et al., 2010). This is consistent with our observation of larger clutch sizes for breeding pairs nesting inside the mammal-exclusion fence. While smaller clutch sizes can result from parents renesting after a failed attempt earlier in the season, we found no difference in clutch sizes at the unfenced site between early and late nesters, suggesting that the smaller average clutch size at the unfenced site compared to the fenced site is not likely due to renesting individuals. However, because most adult stilts were not banded, we were unable to identify if and when individuals renested following a failed nesting attempt. Furthermore, between the sites, there was no detectable difference in the time spent in the nesting area by stilt chicks following hatching. Adult stilts typically lead their broods away from the nest to nearby foraging areas within several days of hatching (Colemann, 1981), potentially to avoid predators that may have discovered the nest site.

When considering the cost effectiveness of mammal-exclusion fencing, conservation planners may also wish to consider the number of species protected, the number of individuals per species protected, and potential gains in reproductive success across species, as well as the trapping effort required to achieve similar gains in reproductive success. The exclusion fence in this study enclosed a relatively small area and protects endangered, native, and migratory species. With a life expectancy of up to 25 years, and a financial break-even point of 16 years compared to trapping alone, fences such as these can be cost-effective over the life of the fence (Moseby & Read, 2006; Sanders, Brown & Keedwell, 2007; Malpas et al. , 2013; Young et al., 2013). The results of this study, demonstrating the substantial reproductive cost of mammalian predators to waterbird nesting success, even with trapping and removal of invasive mammals at the unfenced site, highlight the importance of comparing the effectiveness of potential management actions alongside costs. Future studies should also evaluate the impact of multiple levels of trapping effort at non-fenced sites, to inform comparisons of trapping effort and exclusion fencing, alongside other potential conservation actions (scofield, Ross & Maggie, 2011).

## Conclusions

For conservation-reliant species, particularly those on islands where invasive predator eradication is not currently taking place, conservation fencing may provide a means of increasing nesting success. To our knowledge, our study is the first to evaluate the effectiveness of mammal-exclusion fencing in protecting Hawaiian waterbirds during the breeding season. Our results suggest that mammal-exclusion fencing substantially increases nesting success for at least one endangered waterbird. Further, it suggests that invasive mammals may be the greatest predator-based threat to persistence in this species, as their exclusion from the nesting area resulted in a three-fold increase in the number of chicks hatched per nest.

For other endangered waterbird species that breed year-round inside the enclosure, such as the Hawaiian Coot (‘Alae ke‘oke‘o*; Fulica alai*) and Hawaiian Gallinule (‘Alae ‘ula*; Gallinula galeata sandvicensis*), the recruitment potential could also be substantial. Gains from exclusion fencing may be greater still at sites adjacent to highly urbanized habitats with large source populations of invasive mammals. Exclusion fencing projects may also capture the interest and support of the public, especially communities in close proximity to the refuge (Innes et al., 2012; Innes et al., 2019). As complete island-wide eradication of mammalian predators is not feasible at this time, and endangered Hawaiian waterbirds are conservation-reliant due to impacts from invasive mammals and the necessity of habitat management for nesting success (Reed et al., 2012; Underwood et al. 2013), our study suggests that mammal-exclusion fencing is a highly effective option to increase nesting success in remaining habitat.

##  Supplemental Information

10.7717/peerj.10722/supp-1 Supplemental Information 1 Clean code for publication

10.7717/peerj.10722/supp-2 Supplemental Information 2 2019 Camera Trap Data

10.7717/peerj.10722/supp-3 Supplemental Information 3 2019 Camera Trap Data with Consolidated Fates

10.7717/peerj.10722/supp-4 Supplemental Information 4 USFWS Trapping & Tracking Data (2018–2020)

10.7717/peerj.10722/supp-5 Supplemental Information 5 USFWS Tracking Tunnel Protocol

10.7717/peerj.10722/supp-6 Supplemental Information 6 Aerial image of the unfence Waiawa site and the distribution traps Live traps are labeled with the letter “L” and the kill traps are labeled with a “D” signifying DoC 250 traps.

10.7717/peerj.10722/supp-7 Supplemental Information 7 Aerial image of the fenced Honouliuli site and the distribution traps Live traps are labeled with the letter “L” and the kill traps are labeled with a “D” signifying DoC 250 traps.

10.7717/peerj.10722/supp-8 Supplemental Information 8 Both photos A & B depict the same Hawaiian Stilt nesting pair within the fenced Honouliuli site defending their fresh hatchling, located in the center of the photos, from a Bullfrog (circled in red) approaching the immediate nesting area

10.7717/peerj.10722/supp-9 Supplemental Information 9 Feral cat (circled in red) depredating a Hawaiian Stilt nest at the unfenced Waiawa wetland unit

10.7717/peerj.10722/supp-10 Supplemental Information 10 Histogram (A) displaying hours spent by Hawaiian Stilt chicks in the immediate nesting area at all observed nest sites (X = 57.16; range = 24.5–118.66) and bar plots (B) comparing the hours chicks spent in the nesting area for each site

10.7717/peerj.10722/supp-11 Supplemental Information 11 The measured tracking index for mongoose Squares represent the unfenced site, Waiawa while the triangles and circles represent inside and outside the exclusion fence at Honouliuli, respectively.

10.7717/peerj.10722/supp-12 Supplemental Information 12 The measured tracking index for rats Squares represent the unfenced site, Waiawa while the triangles and circles represent inside and outside the exclusion fence at Honouliuli, respectively.

10.7717/peerj.10722/supp-13 Supplemental Information 13 The measured tracking index for mice Squares represent the unfenced site, Waiawa while the triangles and circles represent inside and outside the exclusion fence at Honouliuli, respectively.

## Additional Information and Declarations

### Field Study Permissions

The following information was supplied relating to field study approvals (i.e., approving body and any reference numbers): All data collection was approved by the U.S Fish and Wildlife Service through a Special Use permit (#2019-006), an Endangered Species Recovery permit (#TE-25955C-2), as well as a state endangered species permit issued by the State of Hawaii Department of Land and Natural Resources, Division of Forestry and Wildlife (#WL-1910).

### Data Availability

The following information was supplied regarding data availability: All code and data files are available at GitHub:
https://github.com/dainlc/Stilt-Survival-PHNWR. Data is also available in the Supplementary Files.
