# Supplementary material for: Mammal-exclusion fencing improves the nesting success of an endangered native Hawaiian waterbird
Source: PeerJ. 2021 Mar 1;9:e10722. doi: 10.7717/peerj.10722 (PMC7931714; doi:10.7717/peerj.10722)
Supplement: Supplemental Information 5 [file peerj-09-10722-s005.docx]

Submitted as a supplemental document for the manuscript:

Christensen, Dain L., Kristen C. Harmon, Nathaniel H. Wehr, and Melissa R. Price. Mammal-exclusion fencing improves the nesting success of an endangered native Hawaiian waterbird. 2020. PeerJ: *In review*

Note: Because of staff shortages and government shutdowns among other reasons, tracking tunnels were not deployed quarterly between the years 2018-2020 as dictated by this USFWS protocol. Due to low sample size and data gaps, tracking indices were inconclusive. Nonetheless, indices were plotted for visual representation (*Supplemental Fig. S6 – S8*).


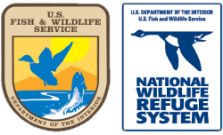


**Site-specific Protocol**

**Predator Control Effectiveness - Non-Native Rodent and Mongoose Relative Abundance (Track Tunnels)**

**FF01RPHB00-033**

**Pearl Harbor NWR**

**Table of Contents**

[1 Introduction 2](#_Toc473710120)

[2 Sampling Design 2](#_Toc473710121)

[3 Field Methods and Sample Processing 3](#_Toc473710122)

[4 Data Management and Analysis 3](#_Toc473710123)

[5 Reporting 5](#_Toc473710124)

[6 Personnel Requirements 5](#_Toc473710125)

[7 Operational Requirements 5](#_Toc473710126)

[8 References 7](#_Toc473710127)

[9 Appendices 7](#_Toc473710128)

# Introduction

## Background

Pearl Harbor NWR was created to provide protected habitat for four species of endemic waterbirds including the Hawaiian duck or koloa maoli (*Anas wyvilliana*), Hawaiian coot or `alae ke`oke`o (Fulica alai), Hawaiian common moorhen or `alae `ula (Gallinula chloropus sandvicensis), and Hawaiian stilt or ae`o (Himantopus mexicanus knudseni), all of which are federally listed as endangered under the Endangered Species Act. Their decline has been attributed to habitat loss and predation by invasive mammalian predators including mongooses, cats, and rats (USFWS 2011). An effective predator control program is critical in ensuring successful reproduction and the continued survival of these species. In recent decades, active control measures for these invasive predators, including fencing, trapping, and rodenticide bait stations, has helped reverse the negative population trends of Hawaii’s waterbirds and populations have stabilized or even increased (Engilis and Pratt 1993, Reed et al. 2011, Underwood et al. 2013).

To evaluate effectiveness of the predator control efforts and support decisions making concerning changes to effort or methods, it is important to conduct periodic monitoring that is independent of the predator removal methods. Tracking tunnel surveys are a common tool used for indexing rodent and mustelid abundance. The survey technique uses a “run through” tunnel containing a pad of paper with ink applied at the center. As an animal passes through the tunnel to investigate bait placed at the center of the card, it picks up the ink on its feet and leaves a set of footprints on the paper as it departs. Tracking tunnels provide an inexpensive and reliable index of the relative abundance of small mammals or any other animal (e.g. large insects, toads, skinks) that can fit through the tunnels. The technique has advantages over live-trapping, including being inexpensive and consistent. It also is non-destructive sampling and does not impact the target population or for that matter any non-target species. Tracking tunnels are also more sensitive than snap traps for detecting the presence of rodents at low abundance (Gillies 2013).

Tracking tunnels only provide a coarse index of relative abundance. They are not a direct measure of population density, but rather provide an index of activity. The technique is best suited for providing simultaneous comparisons of the relative abundance of rodents (particularly rats) or mustelids between similar habitat areas (e.g. treatment and non-treatment) or gross changes in relative abundance over time at a single site. Tracking tunnels are well-suited for monitoring within predator-proof enclosures or monitoring the effectiveness of predator-control efforts.

## Objectives

The survey provides a predator index (mongoose, rats, and mice) that can be tracked over time to determine the effectiveness of ongoing predator control efforts. The survey supports objectives of the refuge’s Comprehensive Conservation Plan (CCP) including:

1.1. Provide seasonal wetland habitat for ae‘o loafing/foraging,

1.2. Manage seasonal wetland habitat for ae‘o breeding,

1.3. Provide seasonal wetland habitat for ‘alae, ke‘oke‘o and ‘alae ‘ula loafing and foraging,

1.4. Provide seasonal wetland habitat for ‘alae ke‘oke‘o and ‘alae ‘ula breeding,

3.1. Conduct inventory and monitoring to document progress and evaluate management strategies, and

3.2. Facilitate research and scientific assessments at the Refuge to guide management decisions.

## Cooperative Surveys

The survey supports the Predator Removal (trapping and removal) survey (FF01RPHB00-040) and is supplemented by a photo monitoring survey (FF01RPHB00-055) to detect cats and other species not identified by tracking tunnels. The tracking tunnel survey is also completed at several other refuges in Hawaii including James Campbell NWR, Kealia Pond NWR, and Kakahaia NWR.

# Sampling Design

## Sample design

The attribute of interest for this monitoring survey is the seasonal relative abundance of mongoose, rats, and mice on the Waiawa and Honouliuli Units of the Pearl Harbor NWR. A predator-proof fence was installed around the Honouliuli Unit in August 2018 and predator eradication efforts have been under way since. The tracking tunnel survey will be used to monitor declines of mongoose, rats, and mice within the enclosure, and subsequently will be used for early detection of predator incursions. The Waiawa Unit is surrounded only by a chain link fence that excludes larger predators, such as dogs and pigs, and humans.

To ensure reasonable accuracy and standardization, tracking tunnel surveys must meet five important assumptions (Gillies 2013): 1) the proportion of tunnels tracked is related to abundance; 2) the relationship between the tracking index and abundance is linear; 3) a constant fraction of individuals is counted between areas at the same time, between areas over time, or within an area over time if survey conditions are standardized; 4) the population must remain demographically closed throughout the survey period; and 5) the selected locations for the tracking tunnels are spatially representative of the habitats within the study area.

A tracking tunnel index is calculated as the percentage of tracking tunnels tracked by each species during the survey. Tracking tunnels only provide a coarse index of relative abundance. They are not a direct measure of population density, rather a measure of activity. The technique is best suited for comparing the relative abundance of predators between similar habitat areas (e.g. treatment and non-treatment) or gross changes in relative abundance over time at a single site (Gillies and Williams 2013). The technique is less useful when target animals occur at very high densities.


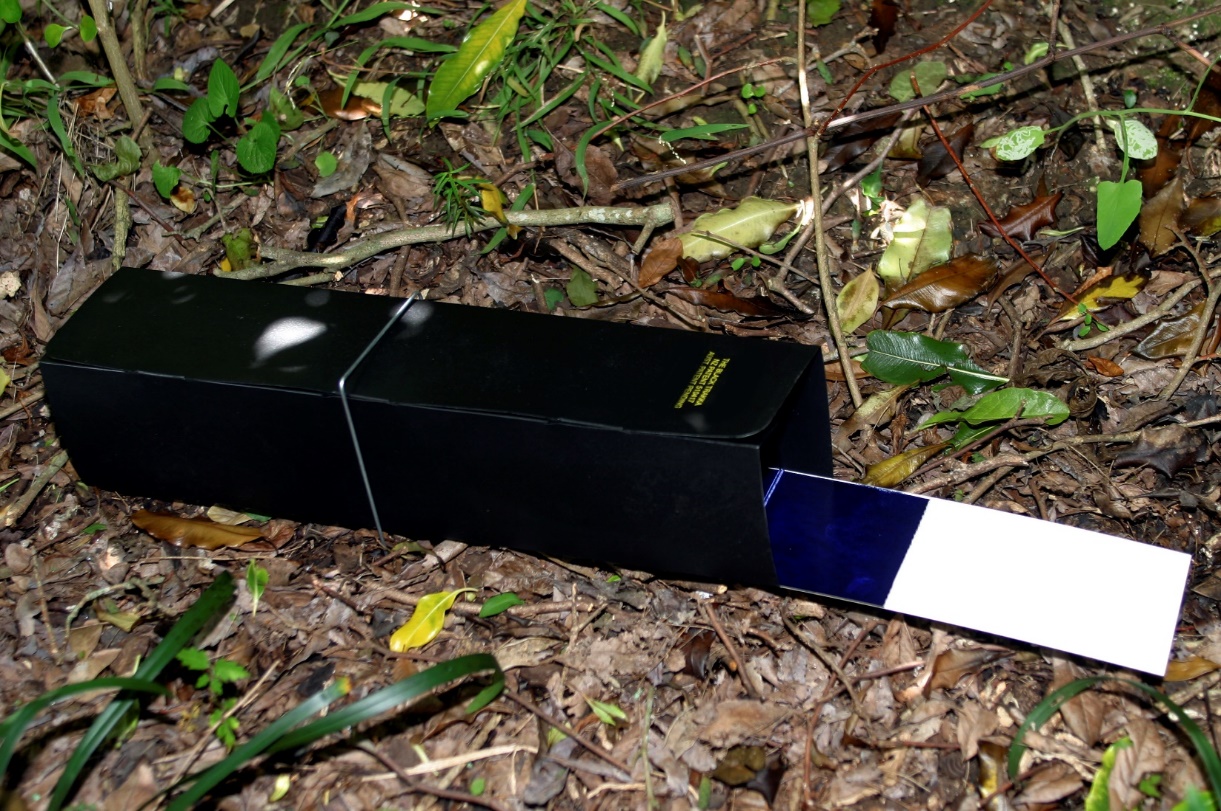


Figure 1. Tracking tunnel with inked tracking card.

## Sampling units

The establishment of sampling units is based on the experiences of Gillies and Williams (2013) for monitoring rodents and mustelids in New Zealand. Survey sampling units are the locations of the tracking tunnels

## Survey Timing

The survey is completed quarterly (January, April, July, October) to better understand the seasonal variation in mongoose and rodent abundance and to assess the effectiveness of predator control efforts.

## Sources of error

The design of a tracking tunnel survey has the inherent disadvantage of not providing an accurate estimate of population size. The survey only provides an index that can be compared over time as the survey is replicated to estimate changes in relative abundance. Also, results should be analyzed carefully since lack of detections does not necessarily mean that animals are not present.

# Field Methods and Sample Processing

## Pre-survey logistics and preparation

### Equipment and supplies

- 32 Tracking tunnels with pegs - <https://www.traps.co.nz/tracking-tunnel-with-card>
- 62 Inked track cards - <https://www.traps.co.nz/tracking-tunnel-cards-spare-154801001>
- Track card ink (optional) - <https://www.traps.co.nz/black-track-ink-dab-ink-pottle>
- Nitrile gloves – to prevent transfer of human scent onto the tunnels and cards and prevent exposure to animal feces.
- Wooden stakes, flagging tape, or other means of marking tunnel locations
- Track Tunnel Maps – (Figure 3 and Figure 4)
- Bait – peanut butter (8 oz. jar) and canned sardines (2 4 oz. cans)
- Hip waders (to access Tunnel HT16 at Honouliuli)

### Refuge access and coordination

- See Appendices 9.1.1 (Waiawa Unit) and 9.2.2 (Honouliuli Unit) for driving directions, gate lock combinations, and special instructions for accessing the refuge units.

### Permits and permissions

This survey is non-intrusive and requires no permits, permissions, or other compliance.

## Establishing sampling units

Using ArcMap, a 100 m fishnet grid was transposed over a map of each refuge unit. Sampling units were placed at the center of each grid square. Some points fell within the wetlands or other locations where placing a tracking tunnel was not possible or practical. These points were moved to the nearest location where a tracking tunnel could be deployed while also maintaining a distance of approximately 100 m from other units and also representing a variety habitats and vegetation types. Usual methods for producing a randomized sampling scheme are inadequate for the Pearl Harbor NWR. The refuge units consist of wetland impoundments surrounded by limited terrestrial habitat, conflicting the placement of tunnels in a grid or line pattern. Also for logistical and safety concerns, it is not practical to place tunnels outside of refuge boundaries.

The Waiawa Unit sampling array includes 10 tracking tunnels (Figure 2) placed in a variety of vegetation types including grass, kiawe forest, near the water edge, and along the fence. The Honouliuli Unit sampling array includes 16 tracking tunnels (Figure 3) placed in various vegetation types, including a tunnel on the small island in the middle of Pond 2. Six additional tunnels were located along the outside of the predator-proof fence. Table 1 includes the Longitude and Latitude for the tunnel locations at each refuge unit. The tracking tunnel locations are marked by wooden stakes painted with blaze orange paint or flagging tape. The tunnel ID is marked on the tunnel and the stake/flagging tape. The tunnel locations are permanent and tunnels are left in place between surveys.

## Data collection procedures

Before conducting the survey (minimum 2 weeks prior) verify that the tracking tunnels are deployed and in serviceable condition. Replace any that have been lost or damaged and ensure that they free of any debris. Ensure the tracking tunnels are secured to the ground using “U” wires designed for this purpose. The best practice is to wear latex gloves when handling the tunnel to prevent the transfer of human scent. Also, verify that the Tunnel_ID (see site maps in appendix) is labelled on the outside of the tunnel using indelible marker. The tunnel locations should also be marked with a highly visible stake or flagging tape to prevent accidental destruction by mowers and vehicles.

### Field Procedures

The survey is conducted over 4 consecutive days.

Day 1 – Label and fill out all information on track card. Place track cards in tunnels and bait each with a ~½” dollop of peanut butter.

Day 2 – Remove the track cards. If tracks are present, fill out and replace with a new card. If tracks are not present, leave original card in tunnel. Remove any remaining peanut butter bait. Rebait the tunnel with a small ~1/2” chunk of sardine.

Day 3 – Do not disturb tunnels and cards.

Day 4 – Remove all track cards from tunnels. Leave tunnels in place if the survey will be conducted again within the next year, otherwise remove them.

Notes: Do not put too much bait on the card or you might draw in too many visits and the card may be difficult to read.

## Processing of collected materials

Examine each card for animal tracks. If tracks are present, they are manually identified by an experienced biologist using available reference materials including Ratz 1997, Russell et al. 2009, and Yuan et al. 2005. Cards that contain tracks but the species cannot be identified are considered “unknown”. A “tracking index” is calculated for each species (i.e. mongoose, rats, and mice) as the number of tunnels with tracks divided by the number of tunnels deployed. If a species is detected on two cards from the same tunnel, then that species is counted just once since this method only discerns presence or absence of species. Track cards are stored in plastic sealable bags in case future analyses are needed. Cards with tracks of “unknown” species should be flagged for future scrutiny.

See appendix for examples of rats, mice, and mongoose found at Pearl Harbor NWR.

## End-of-season procedures

For best results, the tracking tunnels should remain in the field between the quarterly surveys. If the survey will not be completed for at least a year, the tunnels should be removed and stored.

# Data Management and Analysis

## Software

The survey field datasheets and spreadsheet are in Microsoft Excel. No other software is needed to complete the survey.

## Data entry, verification, and editing

1. Open the excel file in the survey folder titled PHR_PredatorControlEffectiveness_TrackingTunnels.xlsx
2. Go to the TrackCard_Data tab and fill out a row of data for each data card. Specific instructions are on the excel sheet.
3. Select RefugeUnit (e.g. PHR_Waiawa, PHR_Honouliuli), date track card was collected, Tunnel_ID, and bait type (typically peanut butter or sardines).
4. Inspect the track card for the each Tunnel_ID. Type the integer ‘1’ into the appropriate column for each species positively identified via tracks on the track card. Leave the columns blank if tracks are not present for that species.
5. If other tracks are present and can be confirmed, for example insects or toads, type the species in the ‘Other’ column.
6. If cards contain tracks that cannot be identified, enter ‘Unknown’ in the ‘Other’ column.
7. Under the 'Comments' column, describe any issues with the track card or other observations.

## Data Analysis

The tracking index is expressed as the mean percentage of tunnels tracked per refuge unit. Regardless of how many tracks a track card may have, cards can only be recorded as tracked or untracked. However, a card can be tracked by multiple species. For example, if both mongoose and mouse tracks are present record the card is tracked by both species.

1. Open the excel file in the survey folder titled PHR_PredatorControlEffectiveness_TrackingTunnels.xlsx
2. On the TrackingIndex_Calculation tab, enter the refuge unit, survey date, total number of tunnels deployed at the unit during the survey, and the total number of the track cards that had positively identified tracks for each species.
3. In Columns G-I, divide the number of tunnels tracked on each by the number of deployed tunnels and multiply by 100 to get the percent of tunnels tracked for each refuge unit and for each species.
4. Chart the Tracking Index versus survey date to show changes over time.

## Data Management and Archiving

The survey data spreadsheet PHR_PredatorControlEffectiveness_TrackingTunnels.xlsx and the survey protocol are located in the Oahu NWR complex shared drive (/REFUGE_SURVEYS/PHNWR/PredatorControl_TrackingTunnels) and on ServCat ().

# Reporting

A summary report should be submitted to the refuge manager within 1 week of completing the survey. The report should include dates the survey was completed, the current tracking tunnel indices for each species (mongoose, rats, and mice), an updated figure showing the tracking indices over time, and any other unique observations including signs of trespass, unique or injured wildlife, or potential safety issues.

# Personnel Requirements

## Roles and responsibilities

The survey can be completed by one individual.

## Qualifications/Training

No special qualifications or formal training are required to conduct the survey. However, identification of tracks may require experienced biologists to ensure accuracy. Surveyors should review useful information in the appendix.

# Operational Requirements

## Safety

- Vagrants and homeless people have been observed accessing the refuge units and the adjacent areas. If any unauthorized people are observed in the refuge unit, do not enter the unit and immediately report the incident to the Refuge Manager. If you are approached by anyone while in the refuge unit or while in-route (e.g. in the residential area, along the bike path), be polite and non-confrontational. If you feel unsafe at any time, leave the area. If you or anyone else are threatened or in immediate danger, call 911.
- Tracked cards and tunnels may become contaminated with animal feces. Always wear latex gloves and thoroughly wash hands and other equipment after handling them.
- Be careful when installing or retrieving the tracking cards as dangerous insects (i.e wasps, centipedes) or other wildlife may be hiding in the tracking tunnels. Inspect the tunnel or give it a good tap or shake before reaching into it.

## Budget

The initial setup costs include deployment and marking of the tracking tunnels in the field. Recurring costs includes the tracking cards and bait for each quarterly survey, as well as estimated replacement costs for lost and damaged equipment.

| **Items** | **Initial setup** | **Recurring (per survey)** |
| --- | --- | --- |
| Tracking tunnels (32) | $250 | $30 |
| Tracking cards (62) |  | $40 |
| Peanut butter (8 oz jar) |  | $3 |
| Sardines (2 cans) |  | $7 |
| Marking sticks (32) | $10 | $3 |
| Marking pen |  | $4 |
| Marking Tape |  | $4 |
| **TOTAL** | **$260** | **$91** |

## Staff time

Estimated staff time does not include time to travel to the refuge units, which will vary depending on the starting location of the surveyor(s). Recurring costs are for each quarterly survey.

| **Tasks** | **Initial setup** | **Recurring (quarterly)** |
| --- | --- | --- |
| Scoping/determine tunnel locations | 4 |  |
| Purchasing supplies | 2 | 0.25 |
| Deploy tunnels | 4 | 2 |
| Day 1 tasks |  | 3 |
| Day 2 tasks |  | 3 |
| Day 4 tasks |  | 2 |
| Track card analysis / ID |  | 2 |
| Data entry and analysis |  | 1.75 |
| Reporting |  | 1 |
| **TOTAL (hours)** | **10** | **15** |
| **FTE (annual)** | **0.005** | **0.029** |

## Schedule

The survey is completed quarterly (January, April, July, and October).

## Coordination

Always coordinate with the refuge manager or biologist when completing the survey. Notify them when you will be accessing the refuge units and keep their contact information with you. Other tasks may be completed on the days of the survey including trap checks or waterbird surveys. This may save other refuge staff from having to travel to the refuge units.

# References

Engilis, A.E. and Pratt, T.K. 1993. Status and population trends of Hawaii’s native waterbirds, 1977-1987. Wilson Bull. 105:142-158.

Gillies, C.A. and Williams, D. 2013: DOC tracking tunnel guide v2.5.2: Using tracking tunnels to monitor rodents and mustelids. Department of Conservation, Science & Capability Group, Hamilton, New Zealand. [www.doc.govt.nz](http://www.doc.govt.nz)

Ratz, H. 1997. Identification of footprints of some small mammals. Mammalia 61(3): 431-441.

Reed, J.M., C. S. Elphick, E. N. Ieno, and A. F. Zuur. 2011. Long-term population trends of endangered Hawaiian waterbirds. Population Ecology 53:473-481.

Russell, J.C., Hasler, N., Klette, R., and Rosenhahn B. 2009. Automatic track recognition of footprints for identifying cryptic species. Ecology, 90(7): 2007–2013.

Underwood, J., Silbernagle, M., Nishimoto, M., and Uyehara, K. 2013. Managing Conservation Reliant Species: Hawai'i's Endangered Endemic Waterbirds. PloS one. 8. e67872. 10.1371/journal.pone.0067872.

U.S. Fish and Wildlife Service. 2011. Recovery Plan for Hawaiian Waterbirds, Second Revision. U.S. Fish and Wildlife Service, Portland, Oregon. xx + 233 pp.

Yuan, G., Russell, J., Rosenhahn, B. and Stones-Havas, S., 2005. Understanding Tracks of Different Species of Rats. CITR, The University of Auckland, New Zealand.

# Appendices

## Site-specific information for Waiawa Unit (PHNWR)

### Waiawa Unit, access instructions


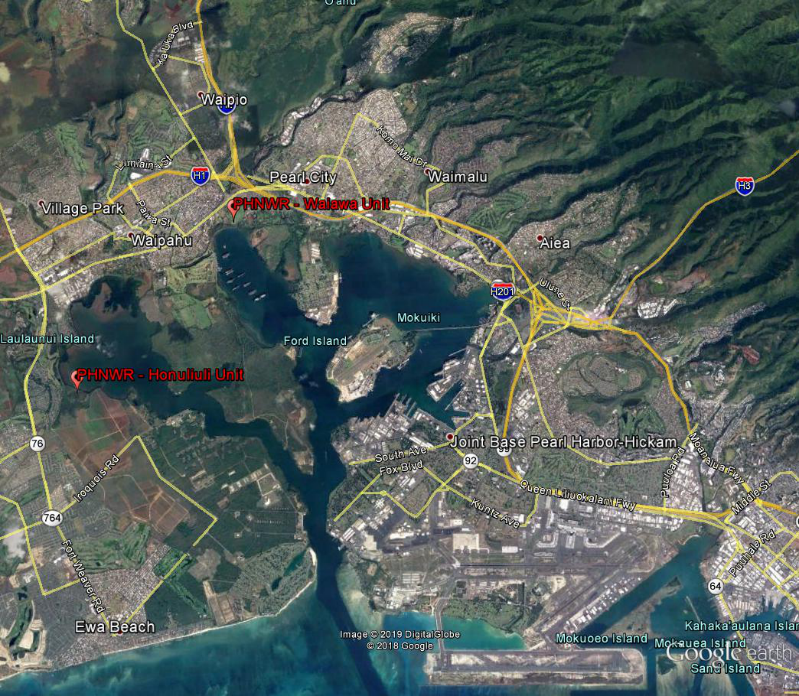


Figure 2. Locations of Pearl Harbor NWR units.

**Directions to Waiawa Unit – from Prince Kūhiō Federal Building (300 Ala Moana Blvd)**

**Google Maps Link:** <https://goo.gl/maps/z1uVMsqeeSFV1sob8>

- Depart the Federal Building and turn south on Punchbowl Street
- Turn RIGHT on Ala Moana Blvd
- Turn RIGHT on Alakea St
- Turn RIGHT on Vineyard Blvd then immediately merge into the far left lane
- Take the next LEFT onto Punchbowl St
- Follow signs and merge onto H1 West (2.2 miles)
- Keep LEFT at the fork to continue on H201 toward Fort Shafter/Aiea (4 miles)
- Use RIGHT 2 lanes to take H1 W toward Pearl City (2.7 miles)
- Take Exit 10 toward Pearl City
- Continue on Moanalua Rd (0.5 miles)
- Turn LEFT onto Ho’olaule’a St (0.5 miles)
- Turn LEFT onto Waimano Home Rd (0.1 miles)
- Turn RIGHT onto HI-99 W (0.5 miles)
- Stay LEFT at the fork to continue on HI-99
- Move to the LEFT lane and take the LEFT turn on Waiawa Rd toward Leeward Community College
- Turn LEFT at the T-intersection and follow Ala Ike to the cul-de-sac
- Take a RIGHT onto Waiawa Rd
- NOTE: Maintain a strict 5 mph speed limit through the residential areas
- Take a LEFT at the fork at … Farms
- Drive slowly down the hill and continue until reaching the Pearl Harbor Bike Path
- Turn RIGHT onto the Bike Path and proceed slowly being cautious of bikers and pedestrians
- The entrance gate to the Waiawa Unit is on the LEFT

**Return to the Prince Kūhiō Federal Building**

- To return to the Federal Building, drive back to Waiawa Rd to the cul-de-sac, drive back toward Leeward Community College via Ike Ake and make the RIGHT turn on Waiawa Rd.
- Take the RIGHT turning lane onto Kamehemeha Hwy (3.2 miles)
- Use RIGHT 2 lanes to turn onto HI-78 E/H201 E (signs for H1 E) (4.9 miles)
- Use RIGHT lane to merge onto H1 E
- Take Exit 21B Punchbowl St. to return to the Federal Building parking lot

**Directions to Waiawa Unit – from Honouliuli Unit**

- Return to Renton Rd and turn LEFT toward Fort Weaver Rd
- Turn RIGHT onto Fort Weaver Rd (~2 miles)
- Take RIGHT ramp onto H1 East toward Honolulu (5.5 miles)
- Take Exit 8A toward Waipahu/Pearl City.
- Merge onto I-99 Kamehameha Hwy
- Keep LEFT at the fork toward H1 E Honolulu and HI-99 E Pearl City
- Move into the LEFT lane and stay left at the fork heading toward HI-99 E Pearl City
- Merge onto HI-99 E/Farrington Rd
- Take RIGHT on Waiawa Rd at the sign for Leeward Community College
- Turn LEFT at the T-intersection and follow Ala Ike to the cul-de-sac
- Take a RIGHT onto Waiawa Rd
- NOTE: Maintain a strict 5 mph speed limit through the residential areas
- Take a LEFT at the fork at … Farms
- Drive slowly down the hill and continue until reaching the Pearl Harbor Bike Path
- Turn RIGHT onto the Bike Path and proceed slowly being cautious of bikers and pedestrians
- The entrance gate to the Waiawa Unit is on the LEFT

**Directions to Waiawa Unit – coming from James Campbell NWR/The North Shore**

- Head SW on HI-83 W
- Continue on HI-83 to HI-80/Kamehameha Hwy (21.6 miles)
- Use RIGHT lane to take H-2 South ramp toward Honolulu (7.9 miles)
- Take Exit 1B for H1 W toward Waianae (0.7 miles)
- Keep RIGHT and follow signs for Waipahu/Pearl City, merge onto HI-99 East Kamehameha Hwy
- Stay LEFT, take exit toward HI-99 E toward Pearl City
- Merge onto HI-99 E/Farrington Rd
- Take RIGHT on Waiawa Rd at the sign for Leeward Community College
- Turn LEFT at the T-intersection and follow Ala Ike to the cul-de-sac
- Take a RIGHT onto Waiawa Rd
- NOTE: Maintain a strict 5 mph speed limit through the residential areas
- Take a LEFT at the fork at … Farms
- Drive slowly down the hill and continue until reaching the Pearl Harbor Bike Path
- Turn RIGHT onto the Bike Path and proceed slowly being cautious of bikers and pedestrians
- The entrance gate to the Waiawa Unit is on the LEFT

**Accessing the Refuge Unit**

- Open the Bike Path gate and drive through, close and lock this gate behind you
- Proceed through the next gate straight ahead and also lock behind you
- The Refuge Unit main gate is to the right. Unlock the gate and drive into the refuge. This gate can remain open while you are in the Unit. Close and lock it when you depart.
- The tracking tunnel system is set up around the perimeter of the refuge unit. You can proceed either clockwise or counter-clockwise around the unit. If the road is too muddy, the survey can be completed on foot.

### Waiawa Unit, tracking tunnel locations


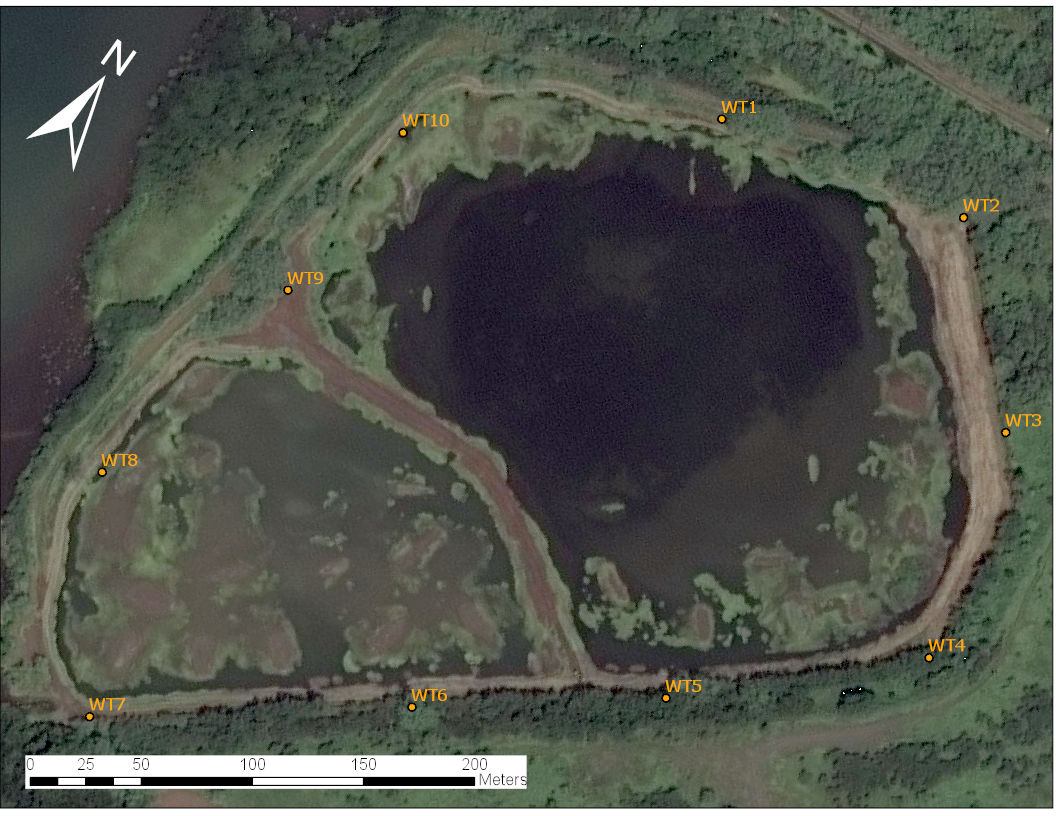


Figure 3. PHNWR, Waiawa Unit - Tracking Tunnel Map

Table 1. PHNWR, Waiawa and Honuliuli Units - Tracking Tunnel Lat/Longs

| TUNNEL ID | LONGITUDE | LATITUDE |
| --- | --- | --- |
| HT01 | -158.021545 | 21.356807 |
| HT02 | -158.021595 | 21.358185 |
| HT03 | -158.020638 | 21.358027 |
| HT04 | -158.019703 | 21.357618 |
| HT05 | -158.019372 | 21.357342 |
| HT06 | -158.01888 | 21.357103 |
| HT07 | -158.017865 | 21.356928 |
| HT08 | -158.01701 | 21.356548 |
| HT09 | -158.017007 | 21.35599 |
| HT10 | -158.017475 | 21.35527 |
| HT11 | -158.01856 | 21.355372 |
| HT12 | -158.01981 | 21.355737 |
| HT13 | -158.020408 | 21.355917 |
| HT14 | -158.020988 | 21.35634 |
| HT15 | -158.021375 | 21.356598 |
| HT16 | -158.019068 | 21.356528 |
| HTO 01 | -158.021707 | 21.358183 |
| HTO 02 | -158.021648 | 21.356765 |
| HTO 03 | -158.020886 | 21.355905 |
| HTO 04 | -158.019529 | 21.355441 |
| HTO 05 | -158.01741 | 21.355116 |
| HTO 06 | -158.016777 | 21.355765 |
| WT1 | -157.98305 | 21.388077 |
| WT2 | -157.98202 | 21.388311 |
| WT4 | -157.981113 | 21.386773 |
| WT3 | -157.981382 | 21.387697 |
| WT5 | -157.981891 | 21.386029 |
| WT6 | -157.982712 | 21.385409 |
| WT7 | -157.983758 | 21.38463 |
| WT8 | -157.984284 | 21.385469 |
| WT9 | -157.984091 | 21.386503 |
| WT10 | -157.984074 | 21.387292 |

## Site-specific information for Honouliuli Unit (PHNWR)

### Honouliuli Unit, driving directions

**Directions to Honouliuli Unit – from Prince Kūhiō Federal Building (300 Ala Moana Blvd)**

- Depart the Federal Building and turn south on Punchbowl Street
- Turn RIGHT on Ala Moana Blvd
- Turn RIGHT on Alakea St
- Turn RIGHT on Vineyard Blvd then immediately merge into the far left lane
- Take the next LEFT onto Punchbowl St
- Follow signs and merge onto H1 West (2.2 miles)
- Keep LEFT at the fork to continue on H201 toward Fort Shafter/Aiea (~5 miles)
- Use RIGHT 2 lanes to take H1 W toward Aiea/Fort Shafter (~8 miles)
- Take RIGHT Exit 5A onto HI-750 S/HI-76 S toward Aiea/Fort Shafter
- Follow HI-76 S/Fort Weaver Rd to the fourth traffic signal. Turn LEFT at Renton Rd.
- Follow Renton Rd for ~1/4 mile. Make the first RIGHT turn onto an unmarked road with a sign reading ‘PRIVATE ROAD Unauthorized Vehicles KEEP OUT’. You are an authorized vehicle and may proceed.
- Keep to the LEFT at the fork. If this road is blocked, the RIGHT fork will lead the same way.
- Proceed through the intersection to the closed gate. This gate has always been unlocked. It opens by moving the slide bar to the left. Pass through the gate and close it behind you.
- Proceed slowly (5 mph) so not to kick up dust and watch out for pedestrians and bikers as the road parallels a bike path. Utility workers are often present on the road and are used to moving out of the way for vehicles accessing the refuge.
- The refuge unit gate is down the road < 1 mile on the RIGHT.
- Open the outer gate and enter the parking lot. Close and lock this gate behind you.
- Open the main gate to the predator-proof fence and drive into the unit. Immediately close and secure the gate behind you.
- When departing make extra sure that the predator-proof fence gate is closed and secured.

**Directions to Honouliuli Unit – from Waiawa Unit**

- Return to Waiawa Rd, the cul-de-sac, and back down Ala Ike toward Leeward Community College.
- Turn RIGHT onto Waiawa Rd and cross straight through the intersection at the traffic signal.
- Turn LEFT

**Directions to Honouliuli Unit – coming from James Campbell NWR/The North Shore**

- Head SW on HI-83 W
- Continue on HI-83 to HI-80/Kamehameha Hwy (21.6 miles)
- Use RIGHT lane to take H-2 South ramp toward Honolulu (7.9 miles)
- Take Exit 1B for H1 W toward Waianae (0.7 miles)

### Honouliuli Unit, tunnel locations


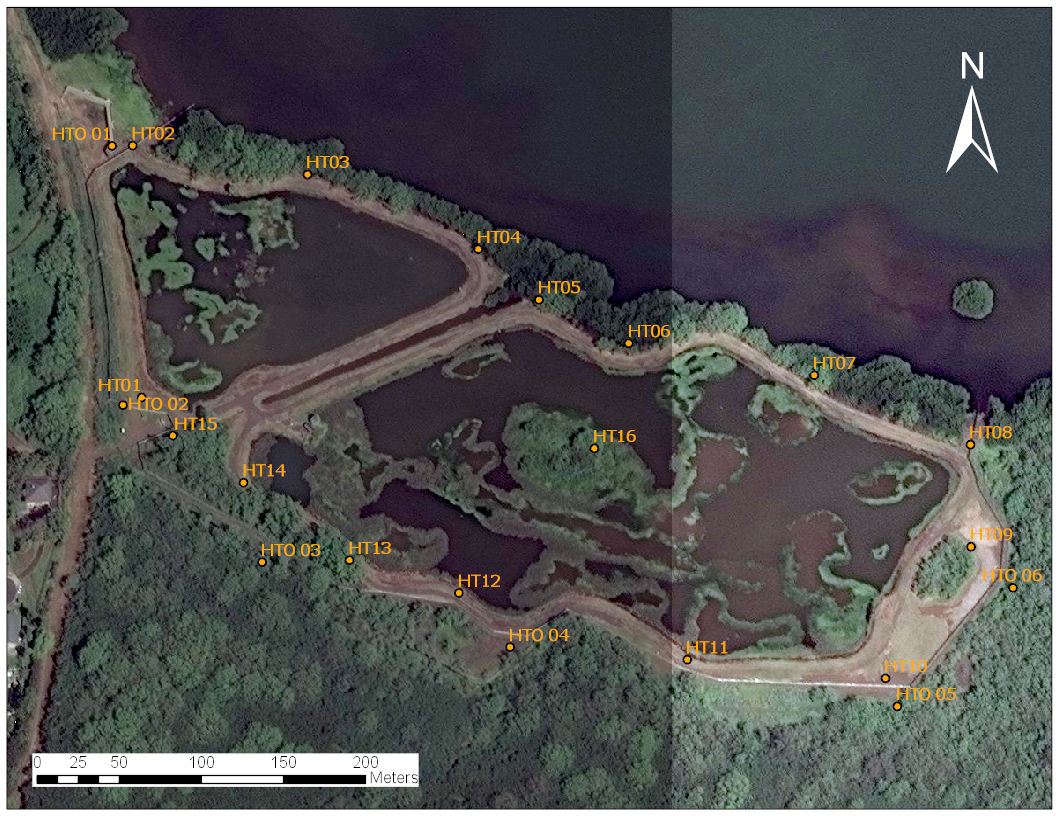


Figure 4. PHNWR, Honouliuli Unit - Tracking Tunnel Map

## Track Card ID examples


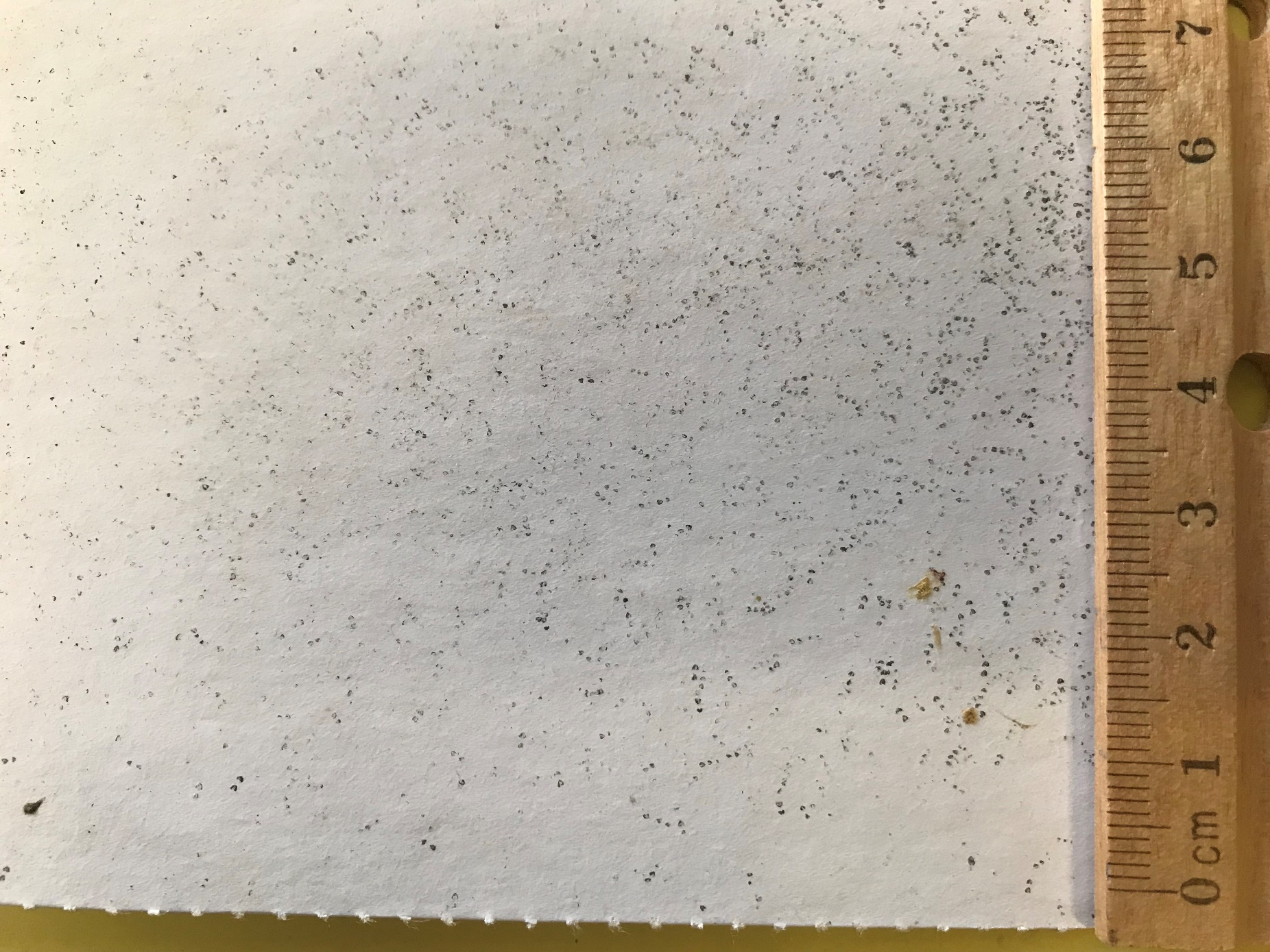


Figure 5. Mouse tracks.


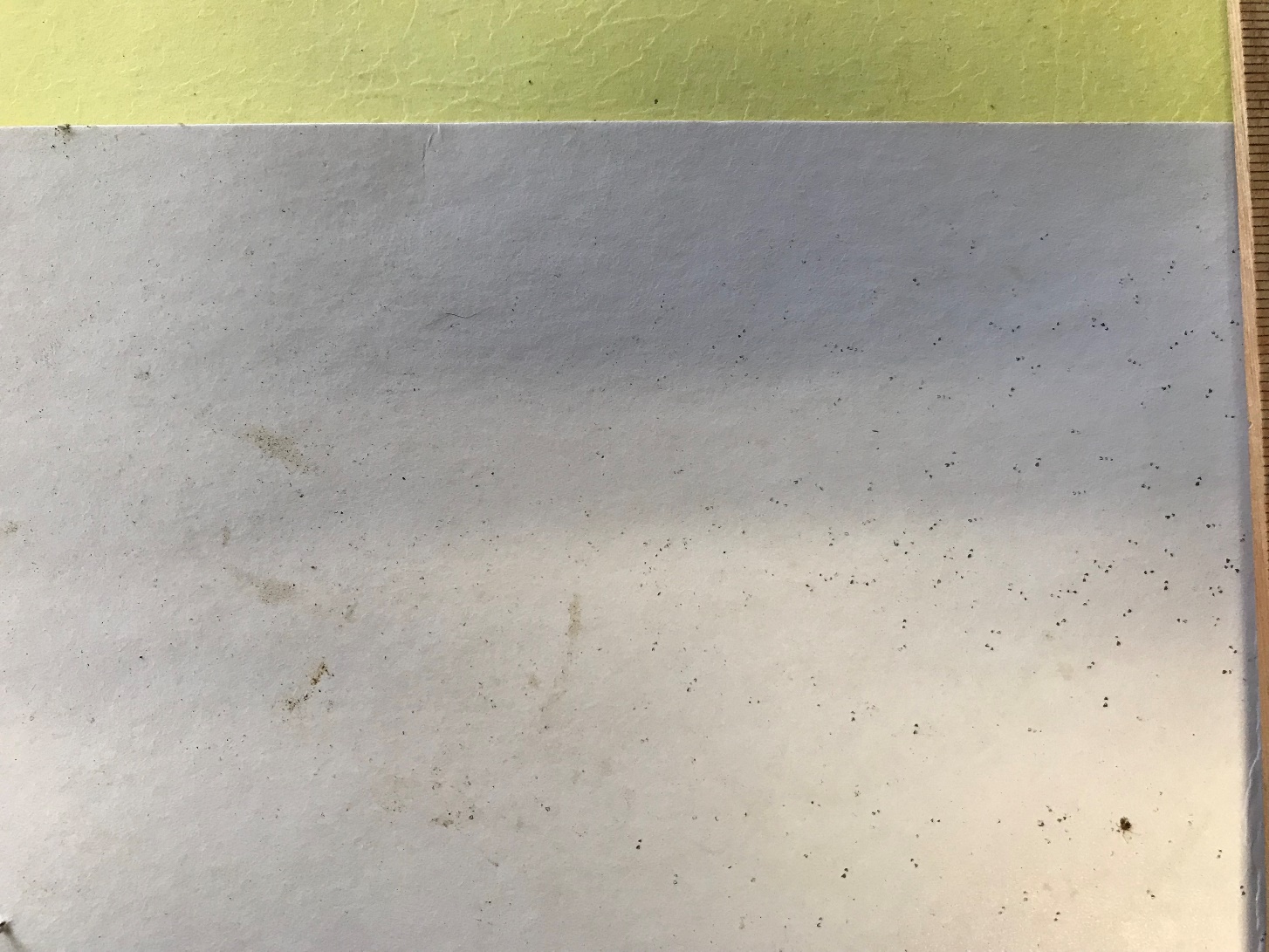


Figure 6. Mouse tracks


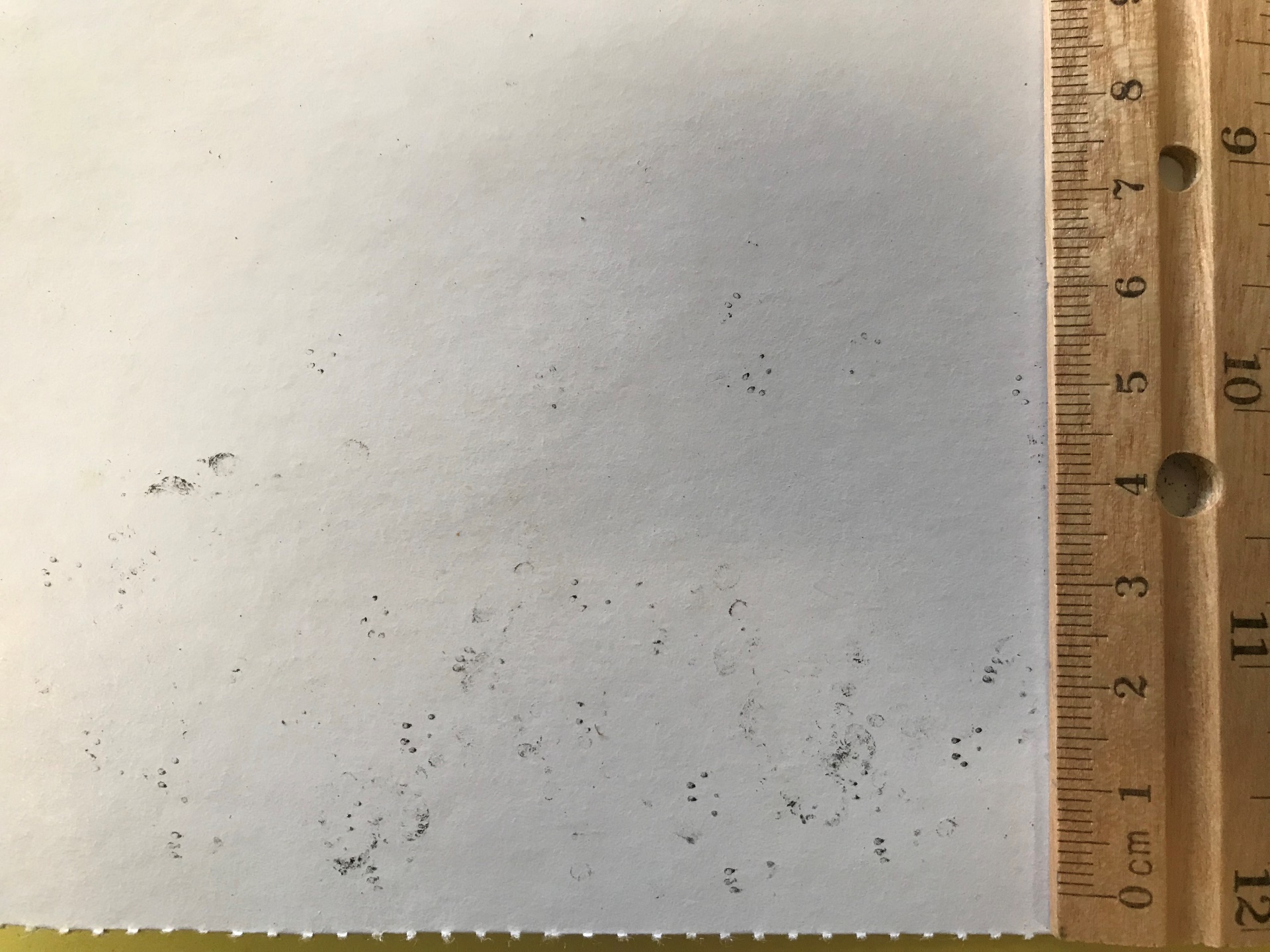


Figure 7. Rat tracks.


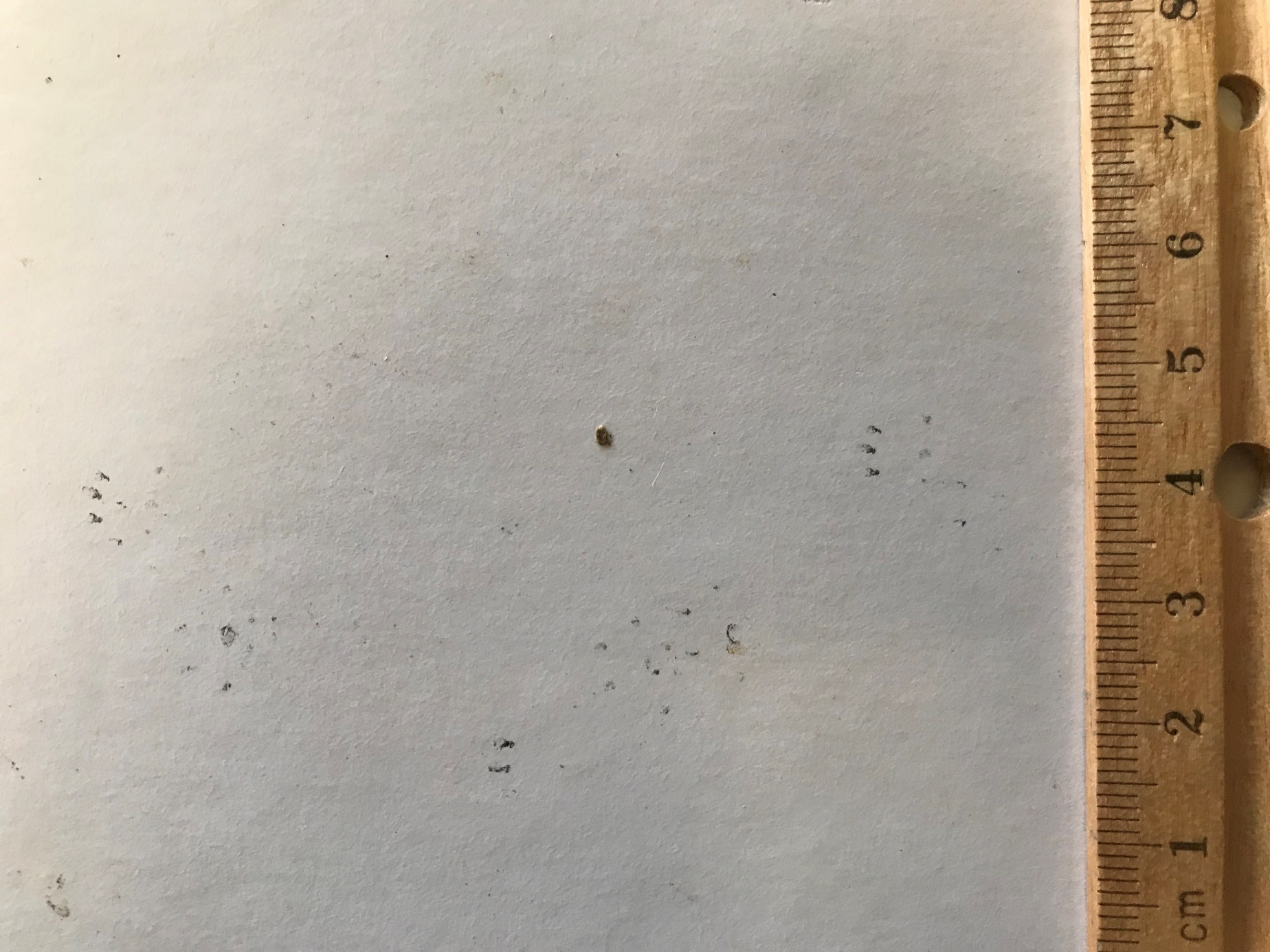


Figure 8. Rat tracks.


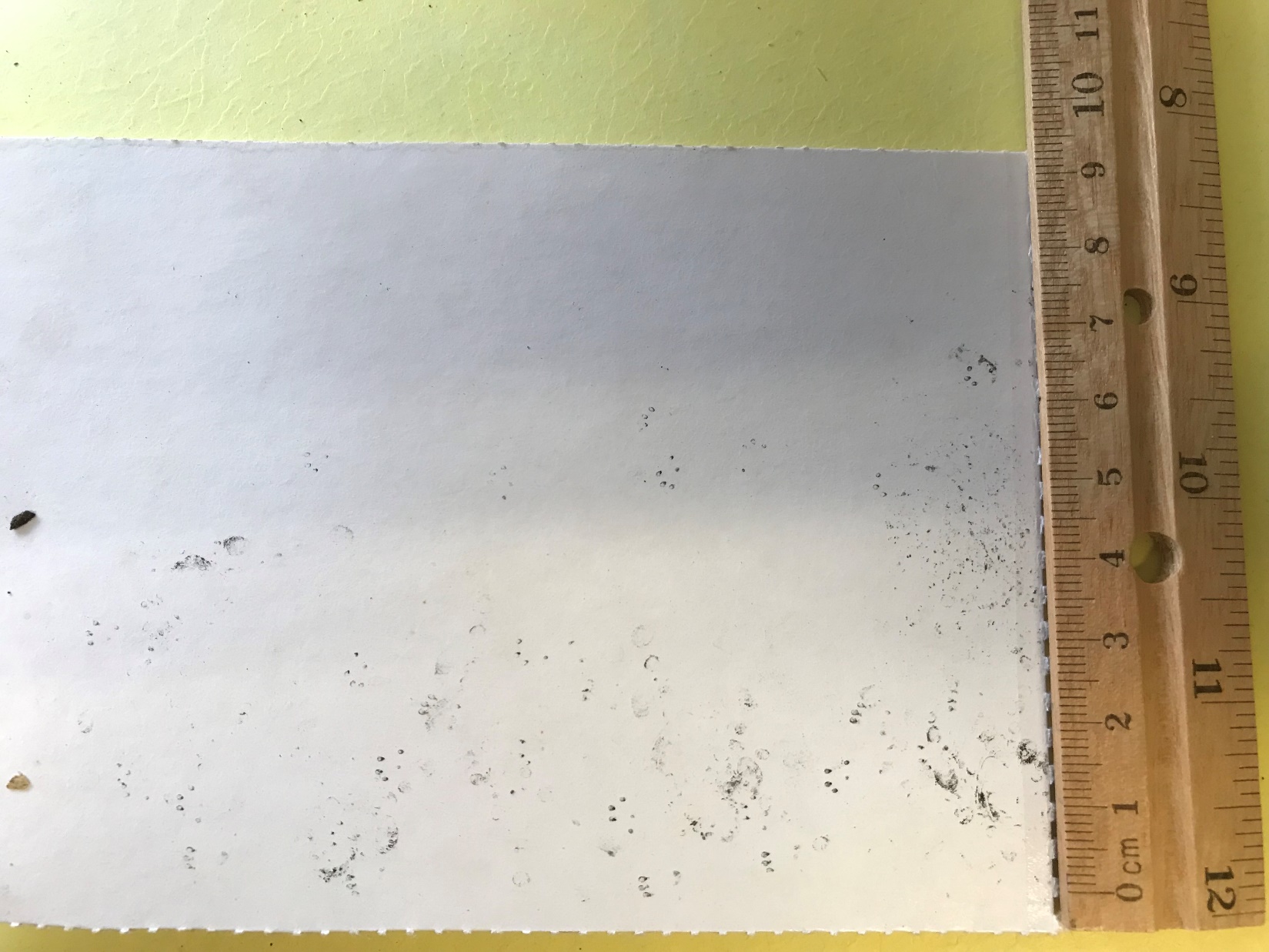


Figure 9. Rat tracks.


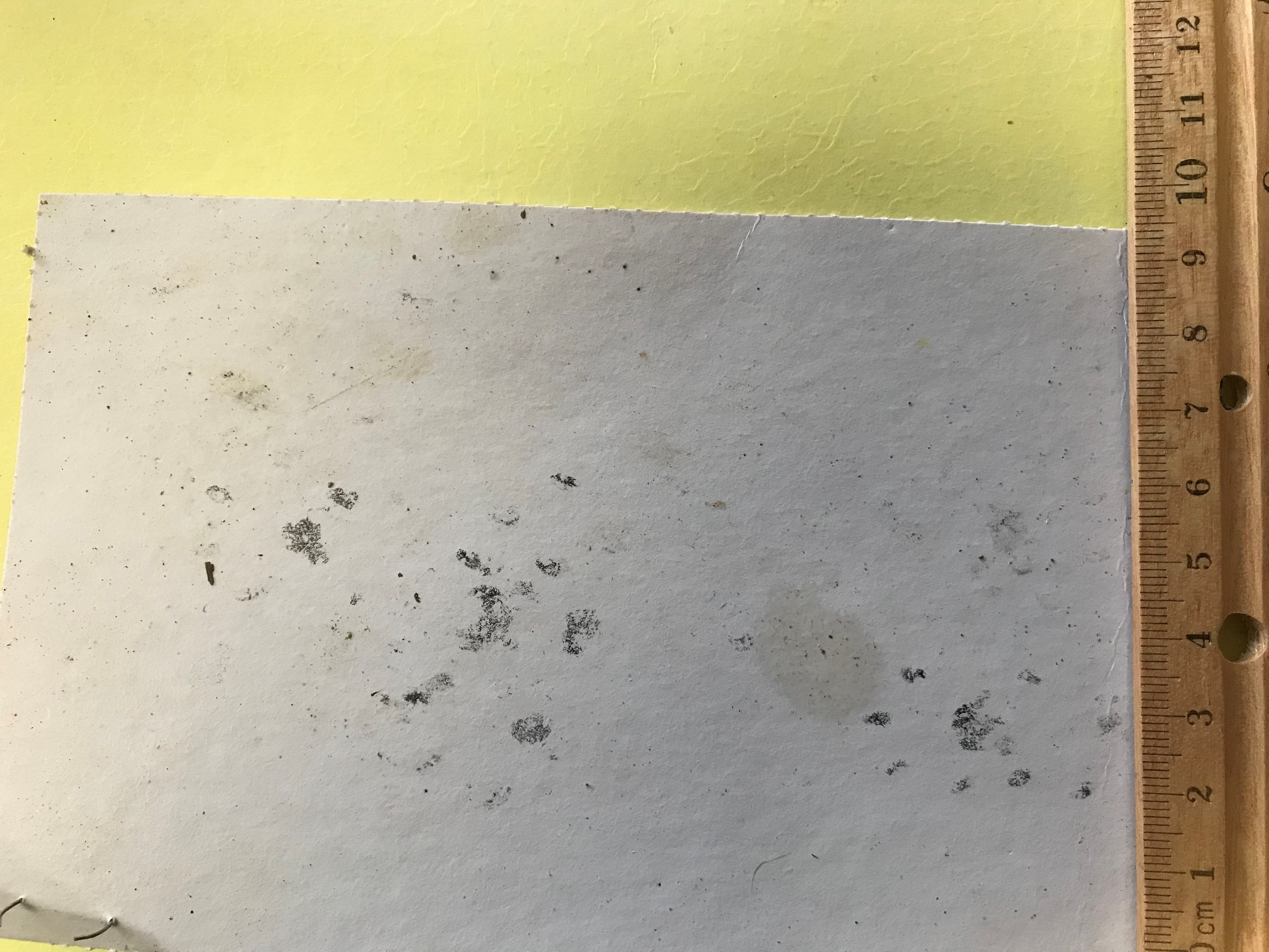


Figure 10. Mongoose and mouse tracks.


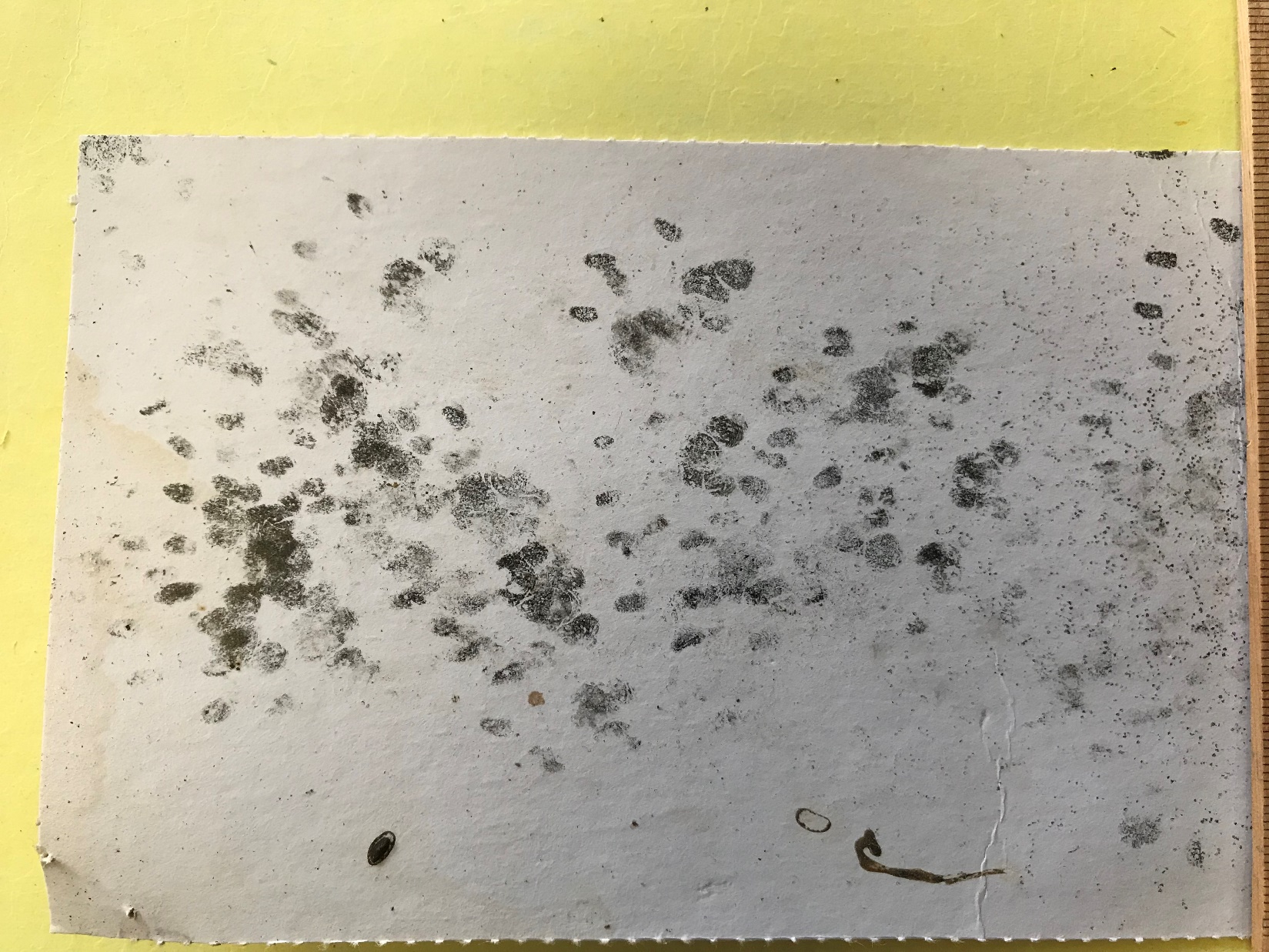


Figure 11. Mongoose and mouse tracks.


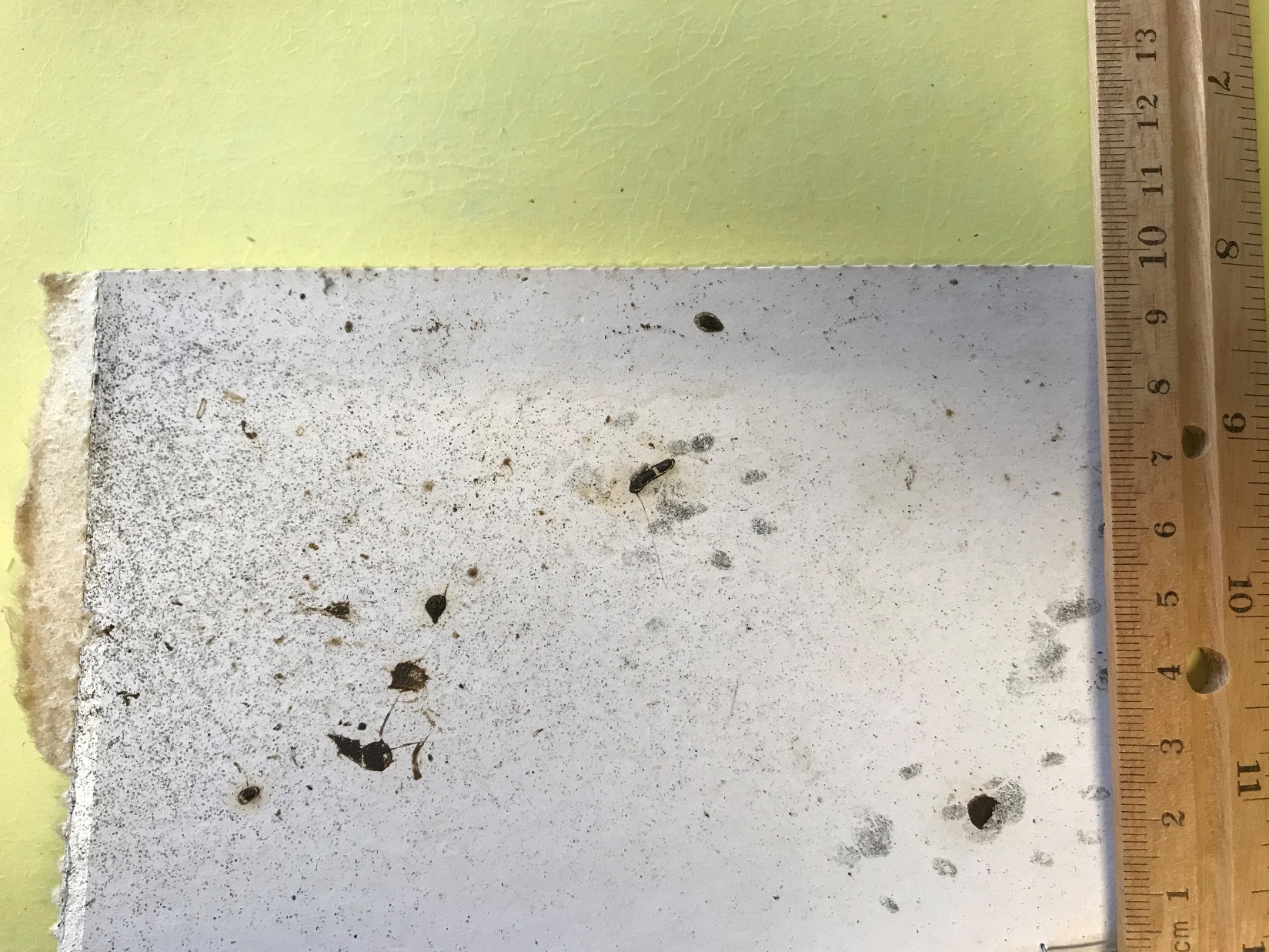


Figure 12. Mongoose and mouse tracks.

## Glossary

## I&M Protocol Review Documentation Form

| **Protocol Title:**  **Version^1^:**  **Date of First Complete Draft: Date of Approval:** | | | | | |
| --- | --- | --- | --- | --- | --- |
| **Refuge Name:** | | | | **Authors and Affiliations** | |
| 'See Survey Protocol Temi;1late instructions on assigning versions. | | | | | |
| **Protocol Type (Select One):**  **A) New Survey Protocol Framework B) Revised Survey Protocol Framework**  **C) New Site-specific Survey Protocol D) Revised Site-specific Survey Protocol** | | | | | |
|  | | | | | |
| **Version** | **Date** | **Author** | **Change Made** | | **Reason for Change** |
|  |  |  |  | |  |
|  |  |  |  | |  |
|  |  |  |  | |  |
|  | | | | | |
| **Internal review(s):** List reviewer comments and describe how they were addressed or why they were not along with each reviewer's name, date review was completed or received, organization, and contact information. If no internal review is used, please briefly describe exemption. Attach separate sheets as necessary. | | | | | |
| **External review(s):** List reviewer comments and describe how they were addressed or why they were not along with each reviewer's name, date review was completed or received, organization, and contact information. If no external review is used, please briefly describe exemption. Attach separate sheets as necessary. | | | | | |

## Appendix D. I&M Protocol Approval Form

| **Protocol Title: Version1 :** | | | | | | | |
| --- | --- | --- | --- | --- | --- | --- | --- |
| **Refuge Name:** | | | | | **Authors and Affiliations** | | |
| **Approvals** | | | | | | | |
| **Action** | | **Signature/Name** | | | | | **Date** |
| **Survey Coordinator ^2^**  **Submitted by:** | |  | | | | |  |
| **I&M Zone Biologist ^3^**  **or equivalent Approval:** | |  | | | | |  |
| **Regional I&M^4^**  **Approval:** | |  | | | | |  |
| **National I&M^5^**  **Approval:** | |  | | | | |  |
|  | | | | | | | |
| **Version** | **Date** | | **Author** | **Change Made** | | **Reason for Change** | |
|  |  | |  |  | |  | |
|  |  | |  |  | |  | |
|  |  | |  |  | |  | |
|  |  | |  |  | |  | |
|  |  | |  |  | |  | |
|  |  | |  |  | |  | |
|  |  | |  |  | |  | |

1 Version number with approval signature at the appropriate level of protocol review.

2 Signature of designated refuge representative for protocols developed and used only at a particular refuge.

**3** Signature signifies approval of site-specific protocols.

**4** Signature by Regional I&M Coordinator signifies approval of protocols used at multiple refuges within a Region.

**5** Signature by National I&M Coordinator signifies approval of protocols used at multiple refuges from two or more

### Version Tracking

*Preparer (name, position, and contact information)*

*Use the table below to track updates to the Survey Methods Record.*

| Version | Completed by | Date | Comments/material updated |
| --- | --- | --- | --- |
|  |  |  |  |
|  |  |  |  |
|  |  |  |  |
|  |  |  |  |
|  |  |  |  |
