# Supplementary figures and images for: Mammal-exclusion fencing improves the nesting success of an endangered native Hawaiian waterbird
Source: PeerJ. 2021 Mar 1;9:e10722. doi: 10.7717/peerj.10722 (PMC7931714; doi:10.7717/peerj.10722)

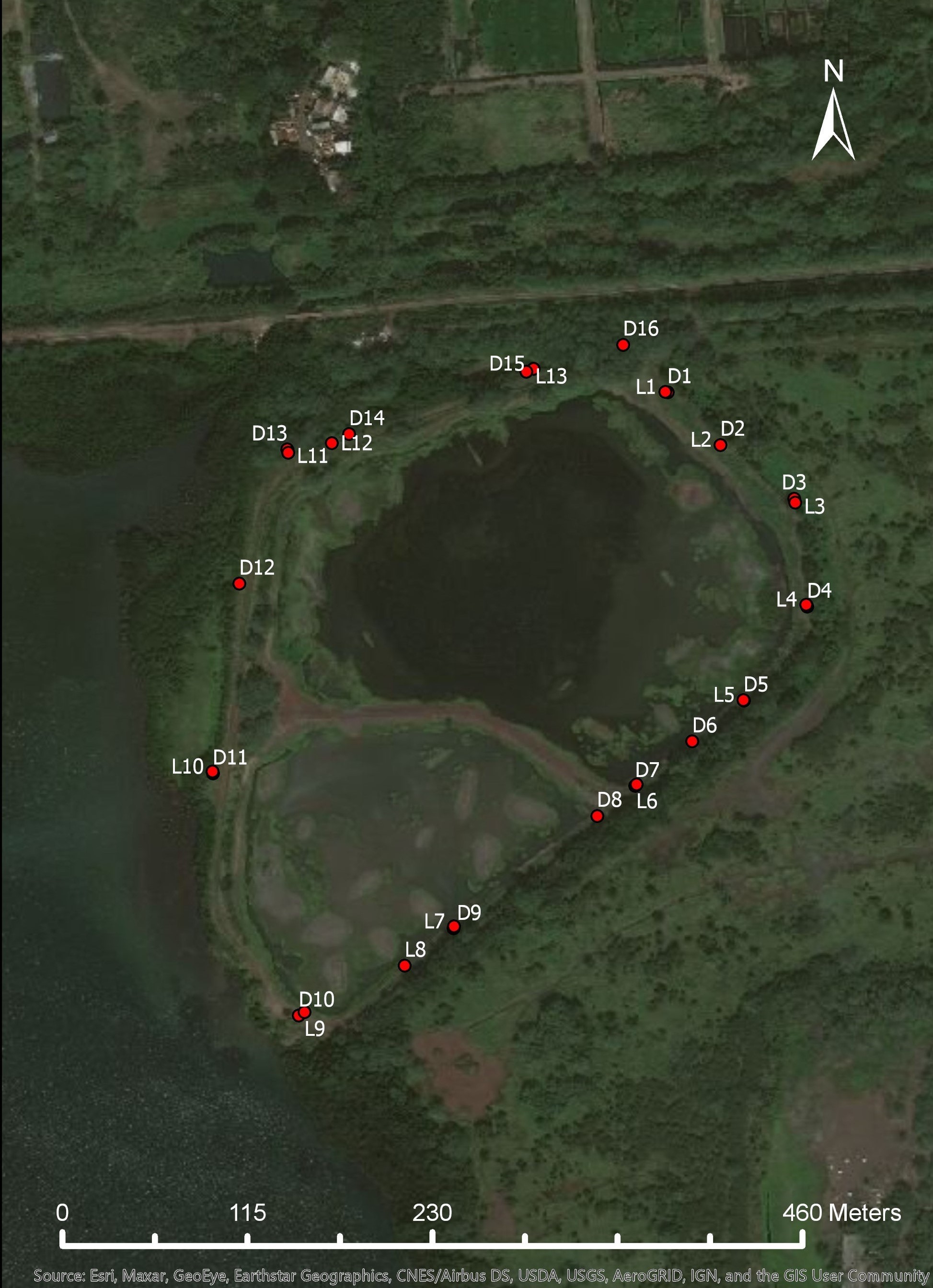

Supplement: Supplemental Information 6 — Live traps are labeled with the letter “L” and the kill traps are labeled with a “D” signifying DoC 250 traps. [file peerj-09-10722-s006.jpg]

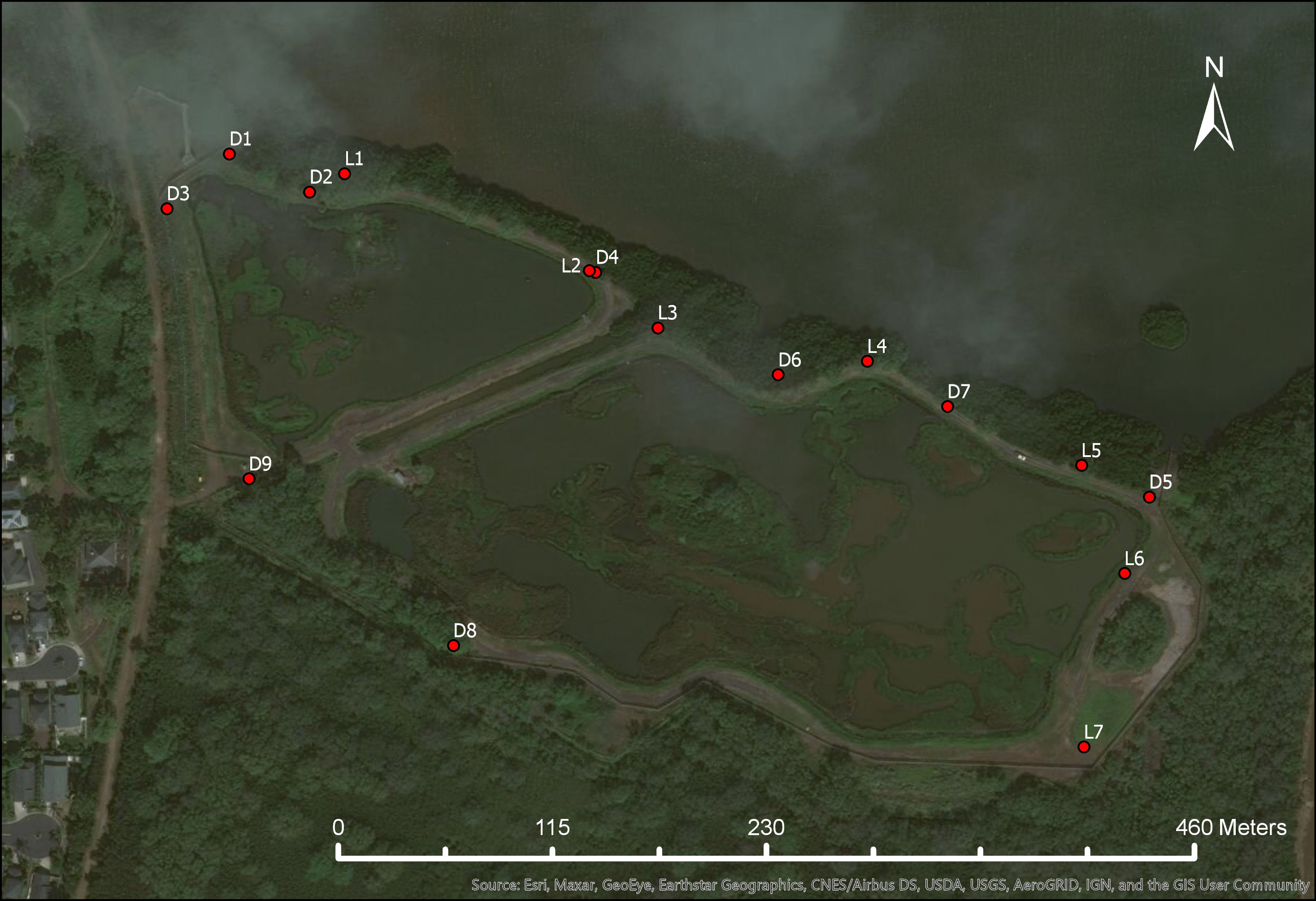

Supplement: Supplemental Information 7 — Live traps are labeled with the letter “L” and the kill traps are labeled with a “D” signifying DoC 250 traps. [file peerj-09-10722-s007.jpg]

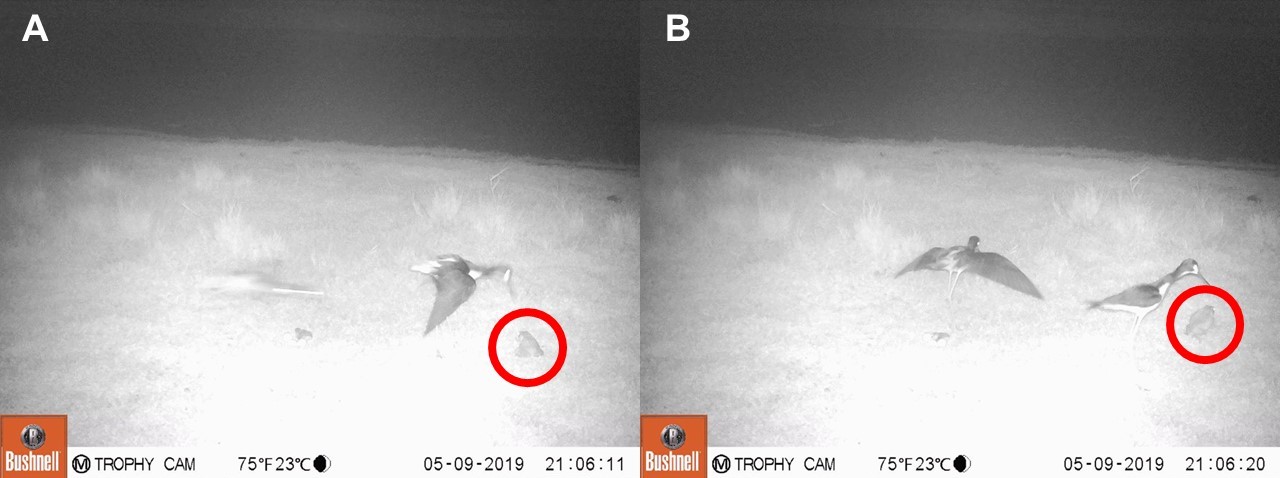

Supplement: Supplemental Information 8 [file peerj-09-10722-s008.jpg]

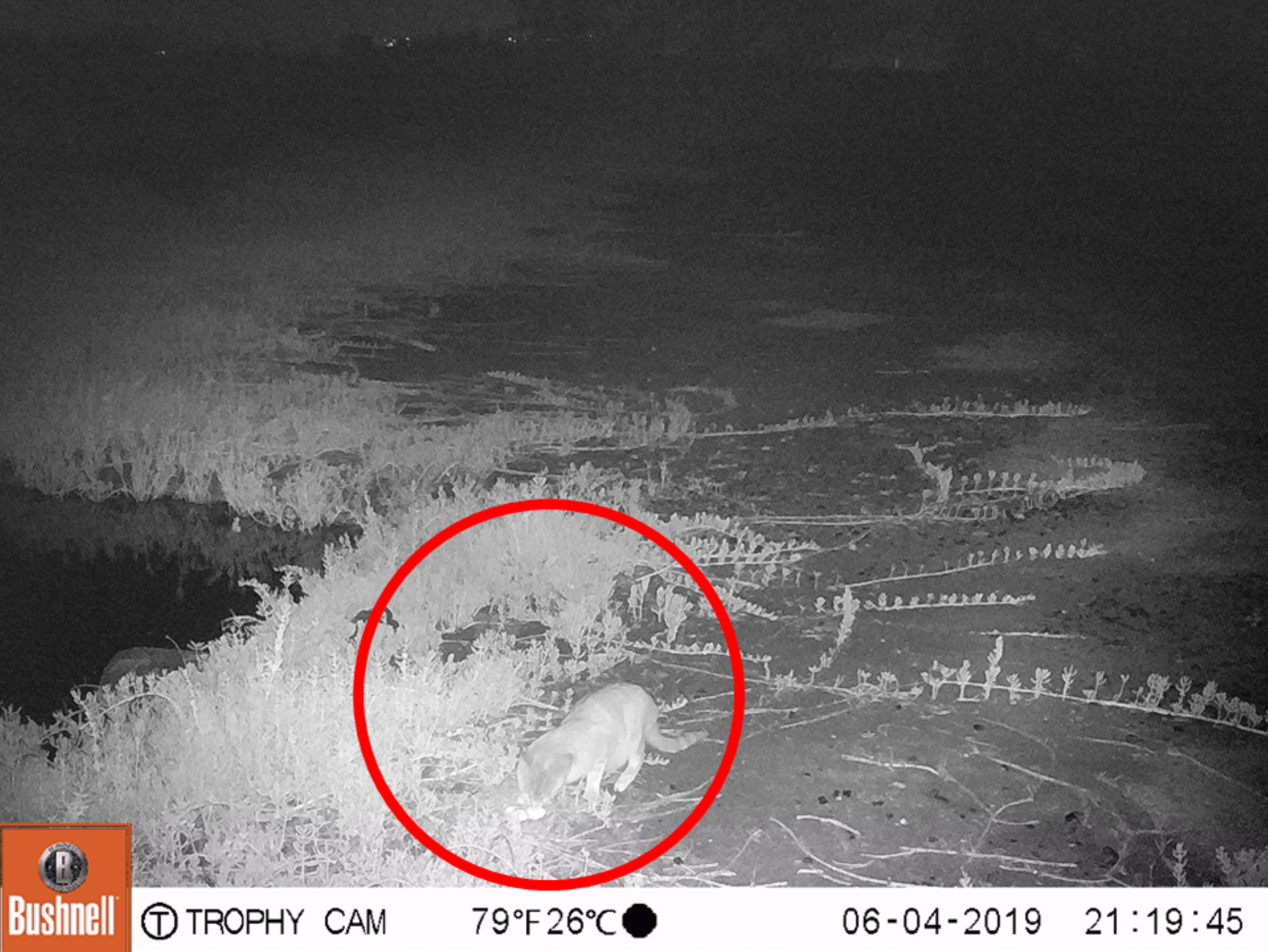

Supplement: Supplemental Information 9 [file peerj-09-10722-s009.png]

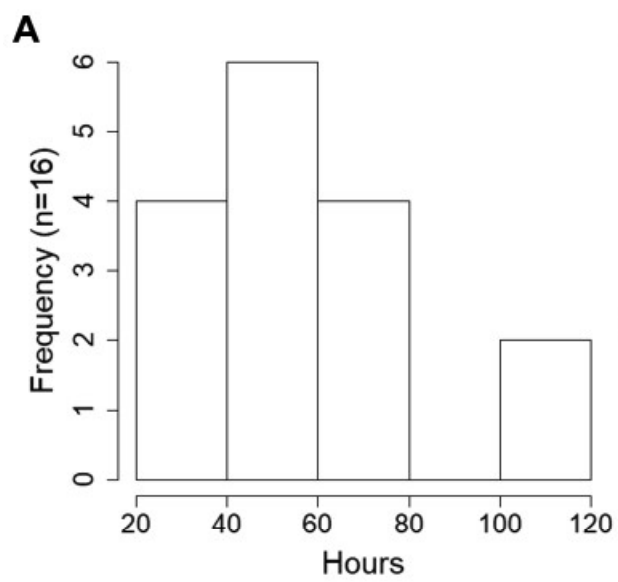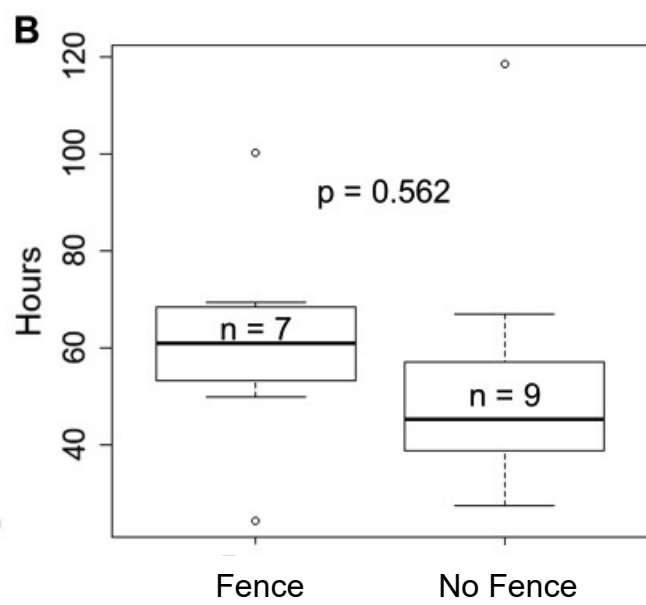

Supplement: Supplemental Information 10 [file peerj-09-10722-s010.pdf]

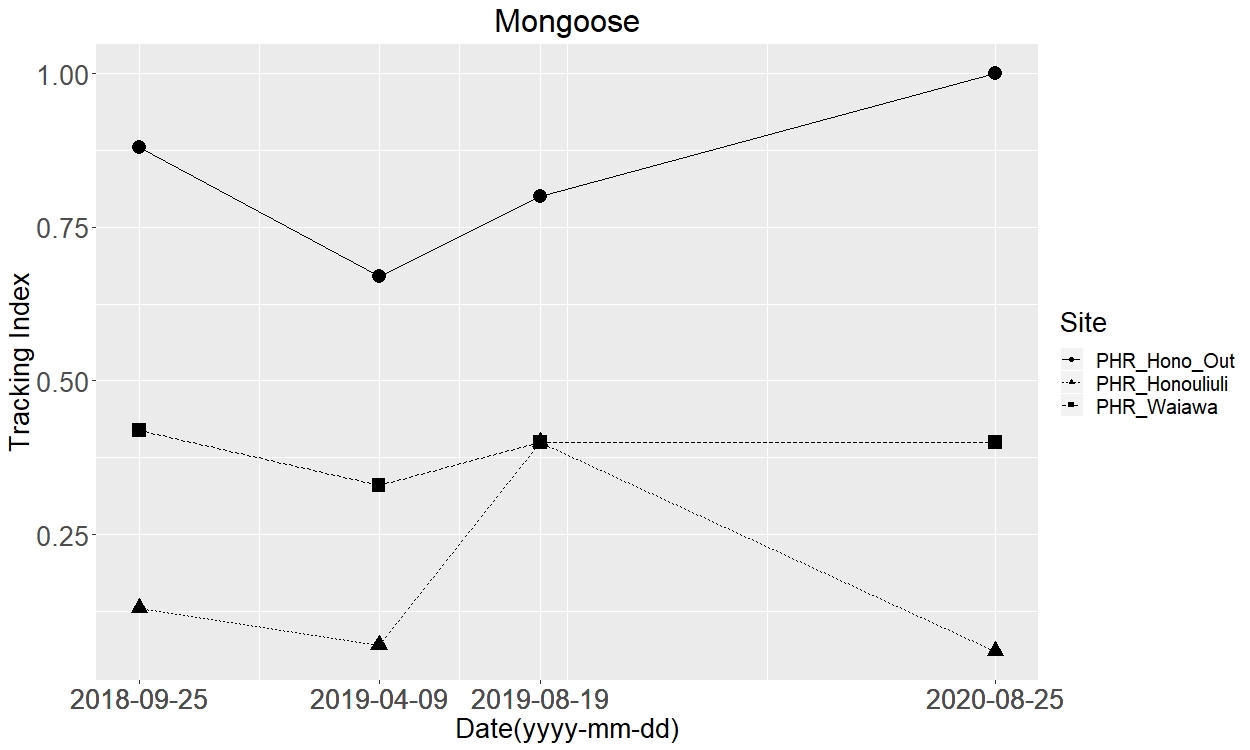

Supplement: Supplemental Information 11 — Squares represent the unfenced site, Waiawa while the triangles and circles represent inside and outside the exclusion fence at Honouliuli, respectively. [file peerj-09-10722-s011.jpeg]

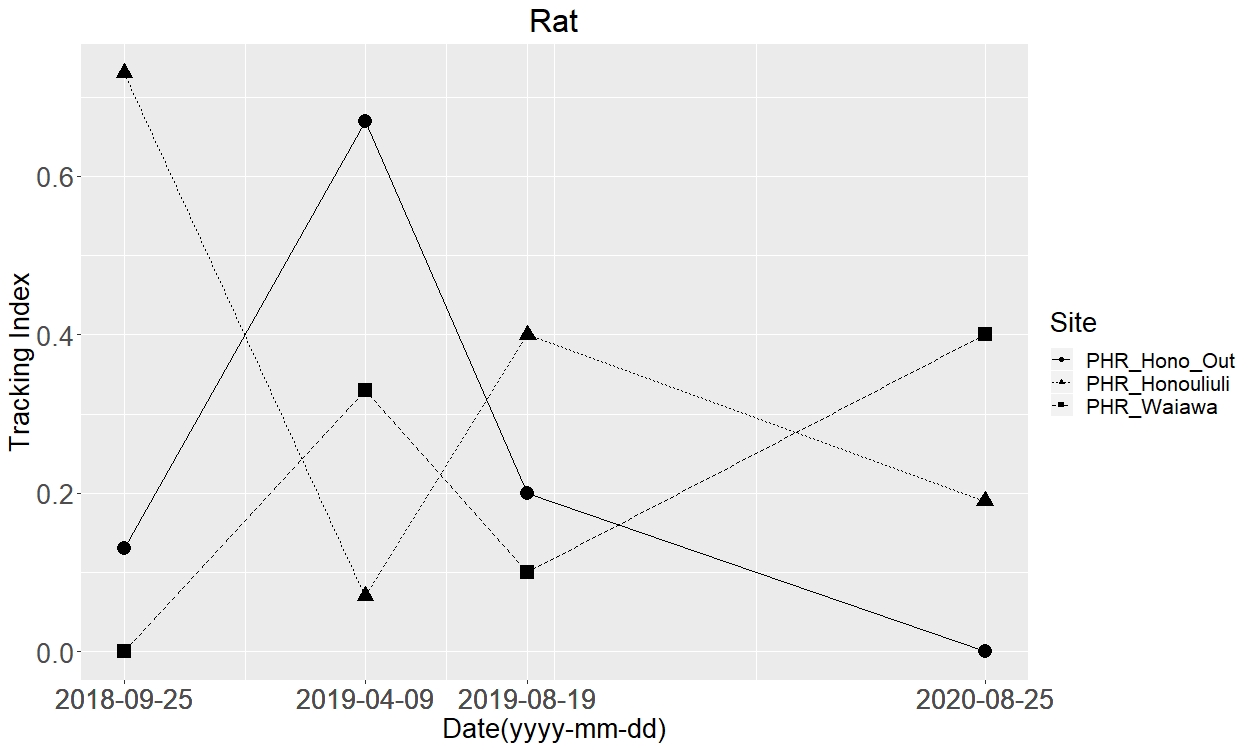

Supplement: Supplemental Information 12 — Squares represent the unfenced site, Waiawa while the triangles and circles represent inside and outside the exclusion fence at Honouliuli, respectively. [file peerj-09-10722-s012.jpeg]

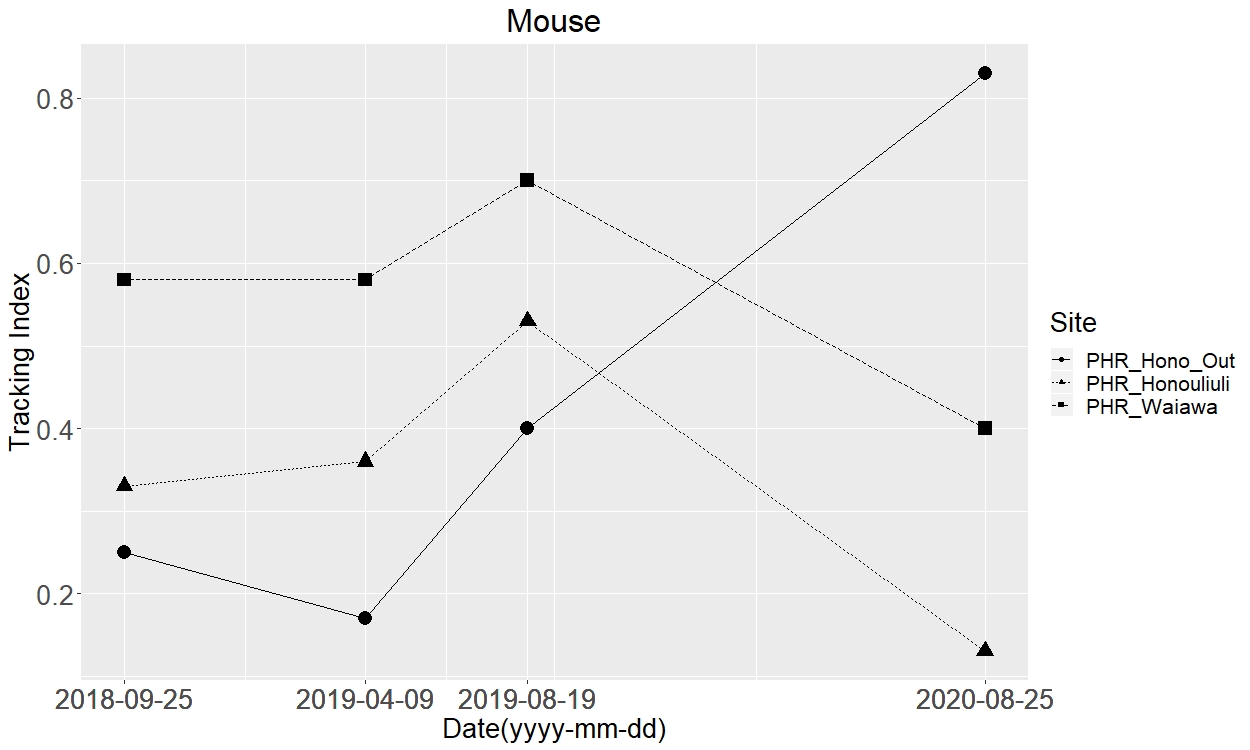

Supplement: Supplemental Information 13 — Squares represent the unfenced site, Waiawa while the triangles and circles represent inside and outside the exclusion fence at Honouliuli, respectively. [file peerj-09-10722-s013.jpeg]
